# Multiple paralogues of α-SNAP in *Giardia lamblia* exhibit independent subcellular localization and redistribution during encystation and stress

**DOI:** 10.1186/s13071-018-3112-1

**Published:** 2018-10-04

**Authors:** Shankari Prasad Datta, Kuladip Jana, Avisek Mondal, Sandipan Ganguly, Srimonti Sarkar

**Affiliations:** 1 Department of Biochemistry, Bose Institute, P 1/12 CIT Road Scheme VII M, Kolkata, West Bengal 700054 India; 2 Division of Molecular Medicine, Bose Institute, P 1/12 CIT Road Scheme VII M, Kolkata, West Bengal 700054 India; 3 Present Address: Section on Developmental Genetics, Eunice Kennedy Shriver National Institute of Child Health and Human Development, NIH, Bethesda, Maryland USA; 4 Division of Parasitology, National Institute of Cholera and Enteric Diseases, P-33, CIT Road, Scheme XM, Beliaghata, Kolkata, West Bengal 700010 India

**Keywords:** α-SNAP, NSF, SNARE, Oxidative stress, Encystation, Functional divergence, Gene duplication

## Abstract

**Background:**

The differently-diverged parasitic protist *Giardia lamblia* is known to have minimal machinery for vesicular transport. Yet, it has three paralogues of SNAP, a crucial component that together with NSF brings about disassembly of the *cis*-SNARE complex formed following vesicle fusion to target membranes. Given that most opisthokont hosts of this gut parasite express only one α-SNAP, this study was undertaken to determine whether these giardial SNAP proteins have undergone functional divergence.

**Results:**

All three SNAP paralogues are expressed in trophozoites, encysting trophozoites and cysts. Even though one of them clusters with γ-SNAP sequences in a phylogenetic tree, functional complementation analysis in yeast indicates that all the three proteins are functionally orthologous to α-SNAP. Localization studies showed a mostly non-overlapping distribution of these α-SNAPs in trophozoites, encysting cells and cysts. In addition, two of the paralogues exhibit substantial subcellular redistribution during encystation, which was also seen following exposure to oxidative stress. However, the expression of the three genes remained unchanged during this redistribution process. There is also a difference in the affinity of each of these α-SNAP paralogues for GlNSF.

**Conclusions:**

None of the genes encoding the three α-SNAPs are pseudogenes and the encoded proteins are likely to discharge non-redundant functions in the different morphological states of *G. lamblia*. Based on the difference in the interaction of individual α-SNAPs with GlNSF and their non-overlapping pattern of subcellular redistribution during encystation and under stress conditions, it may be concluded that the three giardial α-SNAP paralogues have undergone functional divergence. Presence of one of the giardial α-SNAPs at the PDRs of flagella, where neither GlNSF nor any of the SNAREs localize, indicates that this α-SNAP discharges a SNARE-independent role in this gut pathogen.

**Electronic supplementary material:**

The online version of this article (10.1186/s13071-018-3112-1) contains supplementary material, which is available to authorized users.

## Background

*Giardia lamblia* is a gut pathogen that causes the diarrheal disease giardiasis. In addition, this protist serves as an excellent model to study how eukaryotic evolution has proceeded along different paths [1]. Studies show that the molecular machinery for multiple cellular processes of this protist are highly diverged compared to that present in most well-studied model eukaryotes [2]. Whether the differently-diverged cellular pathways of present-day *Giardia* results from early divergence from the main line of eukaryotic evolution or is a consequence of reductive evolution is still debatable. This divergence is particularly evident in the machinery for maintaining its endomembrane system, which is composed of fewer compartments compared to that present in most eukaryotes [2]. Besides the endoplasmic reticulum (ER), the only other identifiable endomembrane compartments are the small peripheral vesicles (PVs) that discharge both endosomal and lysosomal functions [3]. Thus, this parasite is an interesting model to study how vesicular trafficking proceeds in the backdrop of reduced endomembrane compartment diversity and uncovering the molecular machinery that supports this system is important to understand how life has evolved to use different approaches to address the same challenge.

The exchange of material between different endomembrane compartments takes place either through direct contact between the organellar membranes or *via* transport vesicles [4]. Such exchanges are very precise and sustained by a complex machinery whose molecular components include both proteins and lipids [5]. Vesicle-mediated exchange requires membrane deformation and vesicle budding from donor membrane, transport of these vesicles on the cytoskeletal network, followed by docking at the appropriate target membrane, and finally its fusion to the target membrane [6]. Proteins that ensure the fidelity of this process include: (i) the adapter protein (AP) complex and members of the Sar/Arf GTPases that selectively load appropriate cargo molecules into the budding vesicle and recruit the coat proteins at the donor compartment; (ii) the coat proteins (clathrin, COPI and COPII) that deform the membrane to form the vesicle; and (iii) the Rab GTPases, tether proteins and the SNAREs, which ensure that the vesicles only fuse to the appropriate acceptor compartment [7]. While tether proteins extend out from the acceptor membrane to “scout” for the correct vesicle and bring it closer, the SNAREs operate over a shorter distance and the formation of the *trans*-SNARE complex brings about the fusion between the membranes of the vesicle and acceptor compartment.

Different members of the SNARE proteins decorate the surfaces of various vesicles and also the target compartments. A *trans*-SNARE complex is formed when the SNARE on the incoming vesicle precisely pairs-up with the cognate SNAREs on the target membrane. This pairing enables the vesicle and target membranes to come close together such that they can fuse [8]. Following membrane fusion, the helical bundle of the newly-formed *cis*-SNARE complex is disassembled so that the SNAREs arriving on the incoming vesicle may be recycled back to the donor compartment. The strong intermolecular interactions within the *cis*-SNARE complexes are broken with the help of a protein complex, consisting of the AAA ATPase N-ethylmaleimide sensitive factor (NSF) and soluble NSF-attachment protein (SNAP), as the energy derived from ATP hydrolysis drives *cis*-SNARE uncoupling [9].

As previously mentioned, the machinery supporting the endomembrane system of *Giardia* is composed of fewer components. For example, instead of four, only two AP complexes have been identified in *Giardia*; in the tethering complexes, three components of both HOPS and TRAPP1, and two components of DSL1 were found to be missing [10, 11]. Only eight Rab GTPases have been identified in this protist, in contrast to the large repertoire of Rab members in other parasitic protists such as *Trichomonas* and *Entamoeba* [12–15]. Even the ESCRT machinery for endosomal sorting is composed of fewer components, with either entire complexes, such as ESCRT-I, being absent, or complexes being composed of fewer subunits, as in the case of ESCRT-II and ESCRT-III [16, 17].

Interestingly, there appear to be exceptions to this observed reduction of cellular machinery, as in the case of SNAPs. Many organisms, including mammals, have multiple paralogues of SNAPs, termed α-, β- and γ-SNAPs [18]. In mammals, while α- and β-SNAPs share a high degree of homology (> 80% identity), γ-SNAP shares only 20% identity with α-SNAP. Also, while both α- and γ-SNAPs are expressed in most tissues, expression of β-SNAP is restricted to the brain [19]. In addition, expression of α-SNAP commences in the developing embryo and continues into adulthood, but β-SNAP is expressed only after birth [19]. These SNAPs also discharge different cellular functions. α-SNAP is required for transport from ER to the Golgi, within the Golgi, homotypic vesicle fusion, store-operated calcium release and ER/endosome membrane fusion [20–22]. β-SNAP is functionally similar, except it has a different affinity for a binding partner, PICK1 [23]. γ-SNAP can stimulate the Ca^2+^-dependent exocytosis but is unable to function in ER to Golgi transport [24, 25]. While most eukaryotes characterized thus far have only one copy of α-SNAP, two α-SNAPs have been reported in *Giardia* [26]. Here we report the presence of not two, but three SNAP genes in the *Giardia* genome. All the three genes were expressed in trophozoites, encysting trophozoites and cysts. The predicted secondary and tertiary structures of all the three proteins are similar to the yeast α-SNAP, Sec17, and all three giardial genes can rescue the growth defect of the *sec17-1* temperature-sensitive yeast mutant. Interestingly, there is a distinct difference in the subcellular distribution of all three paralogues. Apart from the anticipated localization at the cell periphery where many membranous compartments are present, two of the paralogues exhibit a dramatic change in subcellular distribution both during encystation and oxidative stress. This observed difference in subcellular distribution is indicative of the paralogues performing distinct functions in this protist.

## Results

### *Giardia lamblia* encodes three paralogues of SNAP

**Fig. 1:**
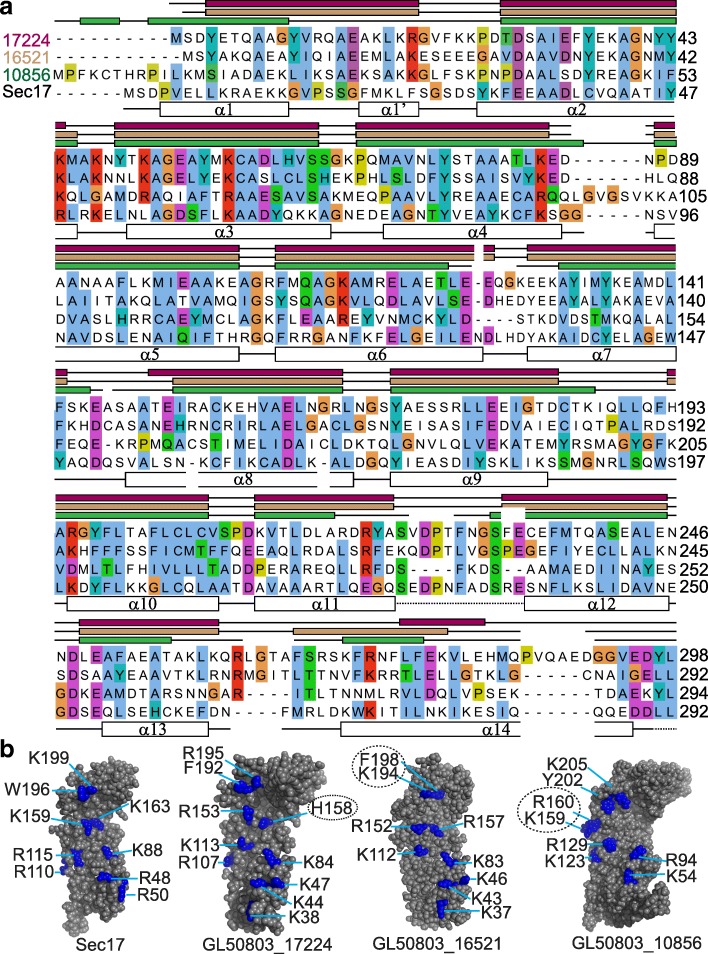
Secondary structural elements and SNARE-binding residues of giardial SNAPs. **a** Sequence alignment of the putative giardial SNAPs with Sec17 of *S. cerevisiae*. α-helical regions of the Sec17 crystal structure (1QQE) have been marked with white boxes below the sequence alignment. The regions of the *Giardia* SNAPs that are predicted to be α-helical have been marked with colored boxes (SNAP_17224_, cherry; SNAP_16521_, fawn; SNAP_10856_, green) above the alignment. Lines indicate loops regions and dashed lines denote the disordered regions in the Sec17 crystal structure. The discontinuity in boxes or lines correspond to gaps in the alignment. **b** Residues that may participate in SNARE binding. Residues, mostly carrying a positive charge (see text), which occupy positions comparable to those of the SNARE-binding residues of the *B. taurus* α-SNAP have been mapped in blue onto the concave face of the threading models of both yeast and *Giardia* SNAPs. Residues that deviate from those of the *B. taurus* α-SNAP, either in terms of charge (GL50803_17224) or position (GL50803_16521 and GL50803_10856), have been marked with dotted circles

Given that SNAPs play pivotal roles in vesicle-mediated trafficking, we wanted to characterize the previously-reported putative giardial orthologues encoded by the ORFs GL50803_17224 and GL50803_16521 [26]. This report also identified the ORF GL50803_10856 as a putative NSF. However, domain analysis in Pfam indicates that like the first two ORFs, the protein encoded by the last also contains a SNAP domain and consistently, GiardiaDB annotates it as an α-SNAP. To determine if the proteins encoded by these ORFs have the potential to function as SNAPs, we analyzed their sequence to determine their probable secondary structures. All characterized SNAP orthologues are small α-helical proteins, including the 292 residue-long *S. cerevisiae* orthologue, Sec17 [27]. The three putative giardial SNAPs are comparable in size; while GL50803_17224 encodes 298 amino acids, GL50803_16521 and GL50803_10856 are composed of 292 and 294 amino acids, respectively. However, the three ORFs do not share extensive sequence homology; while the sequence of GL50803_17224 is 30.6 and 22.6% identical to GL50803_16521 and GL50803_10856, respectively, the remaining two ORFs share 21.6% identity. Secondary structure predictions indicate that all the three putative proteins are composed of α-helices and loops (Fig. 1a). This conclusion is independently supported by circular dichroism spectroscopy, which indicates that the percentage helicity of the three proteins is comparable to Sec17 (Additional file 1: Figure S1). Consistent with the crystal structure of Sec17, the three giardial proteins are predicted to have fourteen α-helices each (Fig. 1a) [27]. One notable difference is that while in the Sec17 crystal structure, the membrane-interacting hydrophobic patch, GFMKLF, adopts a short α turn (α-1’), the hydrophobic residues in the corresponding region of all the three putative giardial orthologues are located in an unstructured loop between helices α1 and α2 [28]. Thus, all the three giardial proteins are like Sec17 both in terms of their size and the secondary structure they are likely to adopt.

A previous report had identified residues on the SNARE-binding surface of the *Bos taurus* α-SNAP, of which all but one (Y200) were charged [29]. Perusal of the crystal structure of Sec17 showed that almost all the analogous positions are also occupied by similar residues, including W196 occupying a position comparable to Y200 (Fig. 1b). Given the low sequence identity of the three giardial proteins with known SNAP orthologues (Additional file 2: Tables S1 and S2), we wanted to determine their possible tertiary structures to see if similarly-charged amino acid residues occupy analogous positions on their surface as well. The possible tertiary structures of the three giardial proteins were determined using protein threading. While most positions on the modeled structures of the giardial SNAPs were occupied by residues analogous to those of the bovine α-SNAP, there were few exceptions: (i) in GL50803_17224, a histidine (H158) was present in place of a canonical arginine or lysine; (ii) there was an interchange of position between an aromatic (F198) and a positively charged residues (K194) in GL50803_16521; and (iii) two positively charged residues (K159 and R160) were shifted more towards the edge of the concave face in GL50803_10856 (dotted circles in Fig. 1b; Additional file 2: Table S3). Barring these minor variations, the pattern of distribution of the positively-charged residues was mostly conserved for all the three giardial proteins and Sec17, indicating that they are likely to be functionally analogous.

**Fig. 2:**
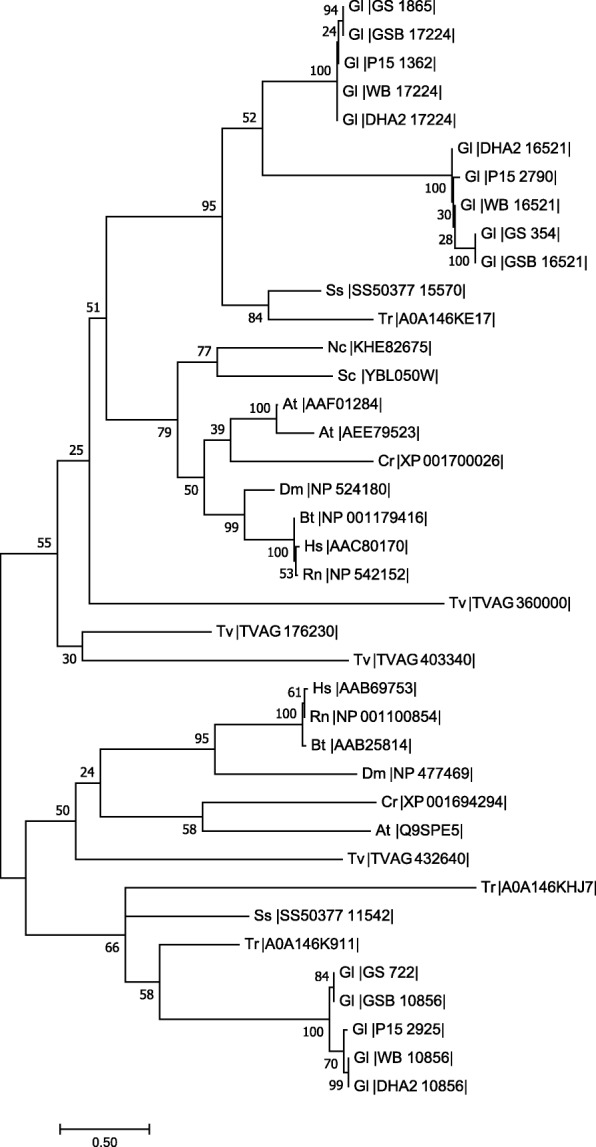
Phylogenetic analysis of the putative SNAPs of *G. lamblia*. Sequences of α- and γ-SNAPs from diverse taxonomic groups, along with those from all five isolates of *G. lamblia* [assemblage A isolate WB (WB), assemblage A2 isolate DHA2 (DHA2), assemblage E isolate P15 (P15), assemblage B isolate GS (GS) and assemblage B isolate GS_B (GSB)] were used to reconstruct a phylogenetic tree using the Maximum Likelihood algorithm. The accession numbers for all homologs used in the analysis are listed next to the organism’s name, while the numerical value next to each node of the phylogenetic tree indicates bootstrap values obtained from 100 replicates. The names of the organisms have been abbreviated as follows: Gl, *Giardia lamblia*; Tr, *Trepomonas* sp.; Tv, *Trichomonas vaginalis*; Ss, *Spironucleus salmonicida*; Nc, *Neurospora crassa*; Sc, *Saccharomyces cerevisiae*; At, *Arabidopsis thaliana*; Cr, *Chlamydomonas reinhardtii*; Dm, *Drosophila melanogaster*; Bt, *Bos taurus*; Hs, *Homo sapiens*; Rn, *Rattus norvegicus*. The scale-bar represents the number of amino acid substitution for each site

Existing literature documents that two of the three α-SNAP proteins, α-SNAP_17224_ and α-SNAP_16521_, are expressed in trophozoites and up to 14 h of encystation [30]. Reverse transcription PCR indicates that all the three identified genes are transcribed in trophozoites, encysting trophozoites (8 and 16 h following induction of encystation), and cysts (Additional file 1: Figure S2). Thus, the identified ORFs are not pseudogenes and the activity encoded by each is required in all stages of this parasite’s life-cycle. Perusal of the genomes of the other *Giardia* isolates included in the GiardiaDB (assemblage A2 isolate DH, assemblage B isolate GS, assemblage B isolate GS_B and assemblage E isolate P15) indicates that each of these also encodes three putative α-SNAPs (data not shown). The presence of multiple α-SNAP paralogues in the genome of a unicellular protist that lacks diversity of endomembrane compartments is unusual given that most eukaryotic genomes encode only one orthologue of this protein; exceptions include *Arabidopsis thaliana*, which has two (Additional file 2: Tables S1 and S2) [31]. To determine if the identified sequences are α- or γ-SNAPs, we reconstructed a phylogenetic tree with known α- and γ-SNAP sequences from diverse taxonomic groups. Sequences of putative SNAP paralogues from the closely-related diplomonad, *S. salmonicida* and *Trepomonas* sp., were also included. From the tree topology, we observed that while two sets of sequences, one with GL50803_17224 and another with GL50803_16521 of the reference strain (assemblage A, isolate WB), cluster together with the α-SNAPs, the third set, with GL50803_10856, clusters closer to the γ-SNAPs (Fig. 2). *Trepomonas*, a close relative of *Giardia*, also contains three SNAP paralogues, of which two cluster with the γ-SNAPs and the third is within the α-SNAP cluster. Unlike *Giardia* and *Trepomonas*, *S. salmonicida* encodes only two putative SNAPs, of which one clusters with the α-SNAPs while the other with the γ-SNAPs. Based on this analysis, it appears that while two of the identified giardial sequences are likely to be α-SNAPs, the third may be a γ-SNAP.

**Fig. 3:**
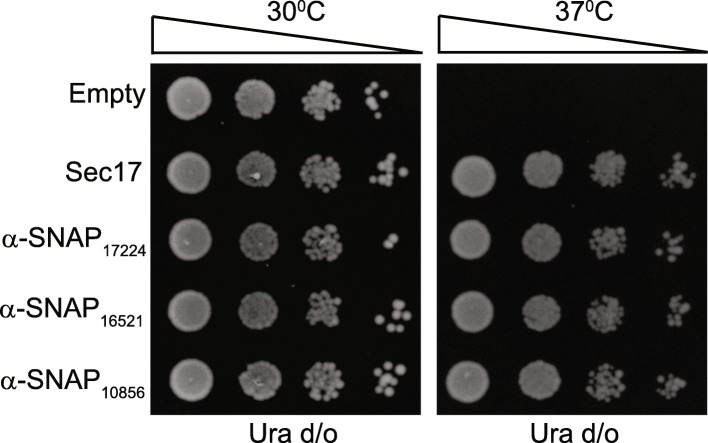
Functional complementation of a temperature-sensitive α-SNAP mutant of yeast with the putative SNAPs of *Giardia*. Temperature-sensitive *sec17-1*mutant yeast strain (RSY269) was transformed with constructs expressing either *SEC17* (positive control) or each of the giardial SNAPs; transformants carrying the empty vector served as negative control. Transformants were spotted onto synthetic medium lacking uracil and incubated at either 30 °C (permissive temperature) or 37 °C (non-permissive temperature)

It is known that α-, but not β- or γ-SNAP can substitute for Sec17 [18]. We used this criterion to evaluate if the identified giardial proteins are α- or γ-SNAP orthologues. Towards this, we used a mutant yeast strain having the temperature-sensitive *sec17-1* allele, which is functional at 30 °C but not at 37 °C [32]. We used functional complementation to assess if any of three giardial genes can functionally substitute for the *sec17-1* hypomorph. All three giardial genes were expressed in the *sec17-1* mutant under the control of a constitutive promoter. Expression of yeast’s own *SEC17* gene from the same vector served as a positive control while yeast transformants containing just the vector backbone served as negative control (Fig. 3). All the five transformants exhibited equivalent growth at the permissive temperature of 30 °C. At the non-permissive temperature of 37 °C, mutants expressing the plasmid-borne copy of *SEC17* exhibited robust growth while those transformants harboring just the vector backbone failed to grow at this non-permissive temperature (Fig. 3). Growth of yeast cells expressing any one of the three giardial genes was comparable to the positive control. Thus, the survival of the temperature sensitive *sec17-1* mutant expressing any of the three giardial genes at 37 °C indicates that even though one of the three SNAP sequences from *Giardia* clusters with γ-SNAPs, all these genes encode proteins that are functionally equivalent to Sec17 of yeast, which is an α-SNAP. Based on the results of this complementation analysis, we conclude that all the three SNAP proteins of *Giardia* are orthologous to α- and not γ-SNAP.

### Unique subcellular localization of the three α-SNAPs indicate functional divergence

The presence of three putative α-SNAPs that are expressed in multiple stages of the parasite’s life-cycle raises the possibility that these may have undergone functional divergence during the course of evolution. Thus, while one of them may be under strong selection pressure to discharge the essential functions of an α-SNAP, the other(s) may have been adapted to perform alternative functions. To investigate if such functional divergence has taken place, we wanted to determine the subcellular distribution of the three α-SNAPs. Towards this, we raised polyclonal antibodies against α-SNAP_17224_ and α-SNAP_16521_ in rabbit and against α-SNAP_10856_ in mouse. Each of the polyclonal antibodies specifically detected only the corresponding protein that had been purified from *E. coli* as a band of ~34 kDa; none of them detected any of the other two α-SNAPs (Additional file 1: Figure S3a). Each antibody also detected a single band in *G. lamblia* trophozoite extract whose size corresponded to that predicted for the α-SNAPs of *Giardia* (Additional file 1: Figure S3a). Thus, in the absence of any observed cross-reactivity, it can be concluded that each antibody recognizes its target α-SNAP with a high degree of specificity. This also indicates substantial structural differences amongst these three proteins.

**Fig. 4:**
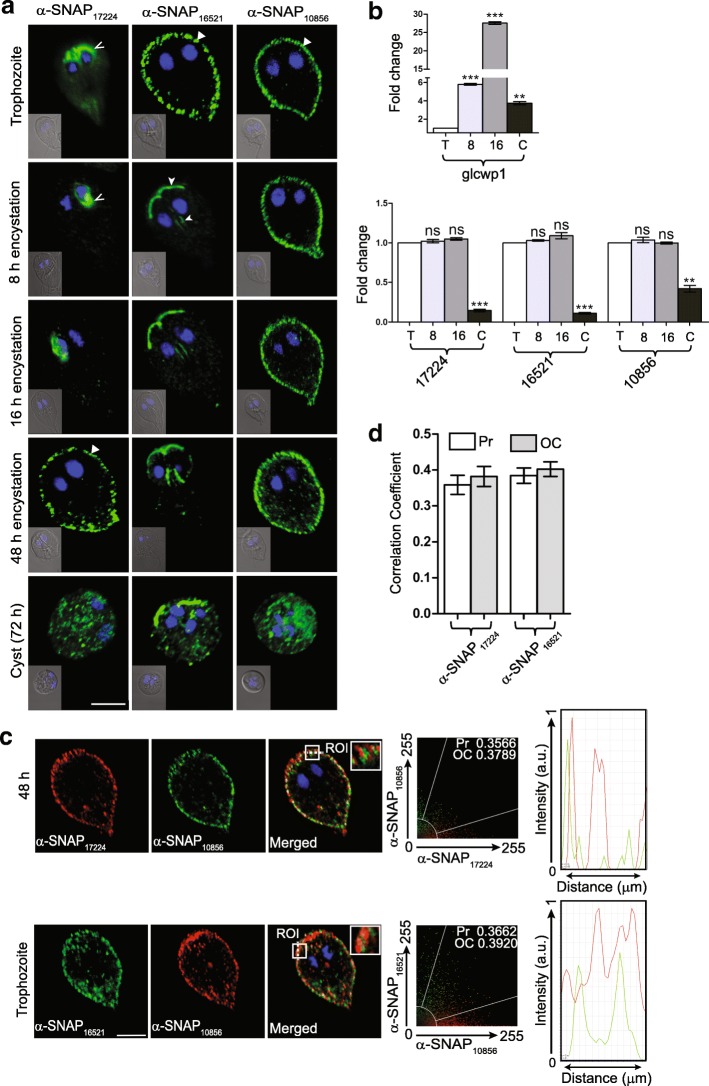
Localization and expression of *Giardia* α-SNAPs in trophozoites, encysting trophozoites and cysts. **a** Immunofluorescence localization, with polyclonal antibodies, of α-SNAP_17224_ (left column), α-SNAP_16521_ (middle column) and α-SNAP_10856_ (right column) in trophozoites, encysting trophozoites (8, 16 and 48 h post-induction of encystation) and cysts. The caret marks the perinuclear region, the arrowhead marks the PDR, and the triangle indicates PVs. To show the localization of α-SNAP_16521_ at both the PDRs and the ventral disc periphery (48 h post-induction), the corresponding panel is an overlay of two z-sections (individual images of the z-stack shown in Additional file 1: Figure S5d). Inset depicts overlay of the DIC and DAPI images. **b** Expression of the α-SNAP genes in trophozoites, encysting trophozoites and cysts were determined by real-time PCR (lower panel), where the expression of CWP1 gene serves as a positive control (upper panel). The asterisks indicate the significance of the difference between the expression under a given condition with that in trophozoites (**P < 0.01; ***P < 0.001; ns, not significant). **c** Colocalization of α-SNAP_17224_ and α-SNAP_10856_ in 48 h encysting trophozoites (top row) or that of α-SNAP_16521_ and α-SNAP_10856_ in trophozoites (bottom row). Insets depict magnification of the region of interest (ROI) that has been marked with a white box. The scattergram in each row indicates the analysis of colocalization between the two fluorophores over the entire z-stack by considering all the pixels within the whole area occupied by that cell. The values for Pearson correlation coefficient (Pr) and overlap coefficient (OC) written inside the scattergrams. The intensity plots at the extreme right indicate changes in fluorescence intensity of the red and green signals across the dotted white line in the ROI. **d** Mean Pr and OC values for multiple z-stacks to determine the extent of colocalization of either α-SNAP_17224_ or α-SNAP_16521_ with α-SNAP_10856_. *Scale-bars*: **a**, **c**, 5 μm

The polyclonal antibodies were used for immunolocalization of the three α-SNAPs in all the different stages of the parasite life-cycle in which their expression had been detected previously (Additional file 1: Figure S2). Given that all three genes can functionally complement the *sec17-1* temperature-sensitive allele (Fig. 3), it is expected that these proteins will be associated with vesicles and/or membrane compartments such as the ER. Consistently, a previous study has already reported the localization of α-SNAP_16521_ to the PVs [33]. We observed that along with α-SNAP_16521_, α-SNAP_10856_ also localizes to the PVs as the signal for this protein colocalizes with that of the fluorescent dye Lucifer yellow, which is endocytosed and delivered to acidic compartments (Fig. 4a; Additional file 1: Figure S4a). α-SNAP_10856_ also colocalizes with the PX domain-containing protein encoded by the ORF GL50803_16548, which is known to localize to the PVs (Additional file 1: Figure S4b) [34, 35]. Unlike the other two, α-SNAP_17224_ localized to the anterior part of the cell, mostly around the two nuclei; another pool of the protein appeared to be cytoplasmic (Fig. 4a). This difference in the subcellular localization between α-SNAP_17224_ and the two other α-SNAP paralogues indicates that the former performs a specific function that is different from that of the other two. Thus, there appears to be a functional divergence for at least one of the three α-SNAPs.

We observed additional evidence of such functional divergence in trophozoites undergoing encystation. While the pattern of localization of α-SNAP_10856_ in trophozoites and encysting cells remained the same, that of the other two paralogues changed significantly (Fig. 4a). At 8 and 16 h after induction of encystation, α-SNAP_17224_ still localized at the perinuclear regions, but its cytoplasmic distribution was no longer evident. Such a distribution persisted even after 30 h of induction (Additional file 1: Figure S5a). However, at 48 h post-induction, it was exclusively located at the PVs, with no signal at the perinuclear region (Fig. 4a; Additional file 3: Figure S9 and Additional file 2: Table S6). α-SNAP_16521_ also underwent a change in cellular distribution during encystation, but this change was much more rapid compared to α-SNAP_17224_. At 8 and 16 h post-induction, α-SNAP_16521_ was present exclusively at the paraflagellar dense rods (PDRs), which are electron-dense structures that are associated with the anterior, caudal and posteriolateral flagella (Fig. 4a; Additional file 1: Figure S5b and c, Additional file 3: Figure S9 and Additional file 2: Table S6) [36]. This transition from the PVs to the PDRs starts as early as 1.5 h after the start of encystation as such cells exhibit both peripheral as well as distribution to the PDRs of the anterior and caudal flagella; complete redistribution of the signal appears to be completed by 4 h (Additional file 1: Figure S5b). The signal was more prominent at the PDRs of the anterior flagella, compared to that of the posteriolateral or caudal flagella. At the 48 h time-point, besides the signal at the PDRs, α-SNAP_16521_ was also observed at the periphery of the ventral disc (Fig. 4a and Additional file 1: Figure S5d). This α-SNAP continued to associate with flagellar structures even in the tetranucleated cysts and also localized to cytoplasmic puncta (Fig. 4a). α-SNAP_17224_ and α-SNAP_10856_ also exhibited a similar punctate distribution in cysts, without any association with the flagella. These puncta are likely to be vesicles as many of these are also positive for the lipid-binding PX domain-containing protein mentioned above (Additional file 1: Figure S6). Although the change in the pattern of subcellular distribution during encystation is unique to each α-SNAP, the pattern of expression of the corresponding genes was very similar during this period. Real-time PCR indicated that the expression of all the three genes remained largely unchanged during encystation, with significant downregulation observed only in cysts (Fig. 4b, lower panel). The expression of the gene encoding CWP1 served as a positive control (Fig. 4b, upper panel) [37]. Such subcellular redistribution of proteins, without any change at the level of transcription, has been previously observed for other giardial proteins, such as β’COP subunit, Rab11, YiP, heavy chain of clathrin, DRP, ESCP and Rpn10 [13, 38–40]. Thus, many regulatory changes in this protist appear to be dependent on protein relocation, rather than synthesis of new proteins.

There are two situations in which two of the α-SNAPs exhibit a similar peripheral distribution in trophozoites or encysting trophozoites: (i) α-SNAP_16521_ and α-SNAP_10856_ in trophozoites and (ii) α-SNAP_17224_ and α-SNAP_10856_, 48 h post-induction of encystation (Fig. 4a). To determine if the two above-mentioned protein pairs colocalize, we carried out quantitative colocalization analysis for each pair under the conditions in which they exhibit similar cellular distribution. Scattergrams of multiple images of both the protein pairs did not indicate any substantial colocalization as the distributions of the green and red-colored pixels did not overlap significantly (Fig. 4c). Both the scattergram and the intensity plot of the two different fluorophores indicate that while there was some overlap for α-SNAP_16521_ and α-SNAP_10856_ in trophozoites, the same was not observed for α-SNAP_17224_ and α-SNAP_10856_, in encysting trophozoites (Fig. 4c). This is supported by colocalization analysis of multiple images wherein both the Pearson’s correlation (Pr) and overlap coefficient (OC) values are below 0.5 for each protein pair (Fig. 4d). Incidentally, SNAP_16521_ and α-SNAP_17224_ never localize to the same subcellular region under any of the conditions tested (trophozoites, encysting trophozoites and cysts) (Fig. 4a). Given this lack of colocalization of the three α-SNAP paralogues, it may be concluded that there are functional differences among these three proteins.

### All the α-SNAP paralogues colocalize with NSF

**Fig. 5:**
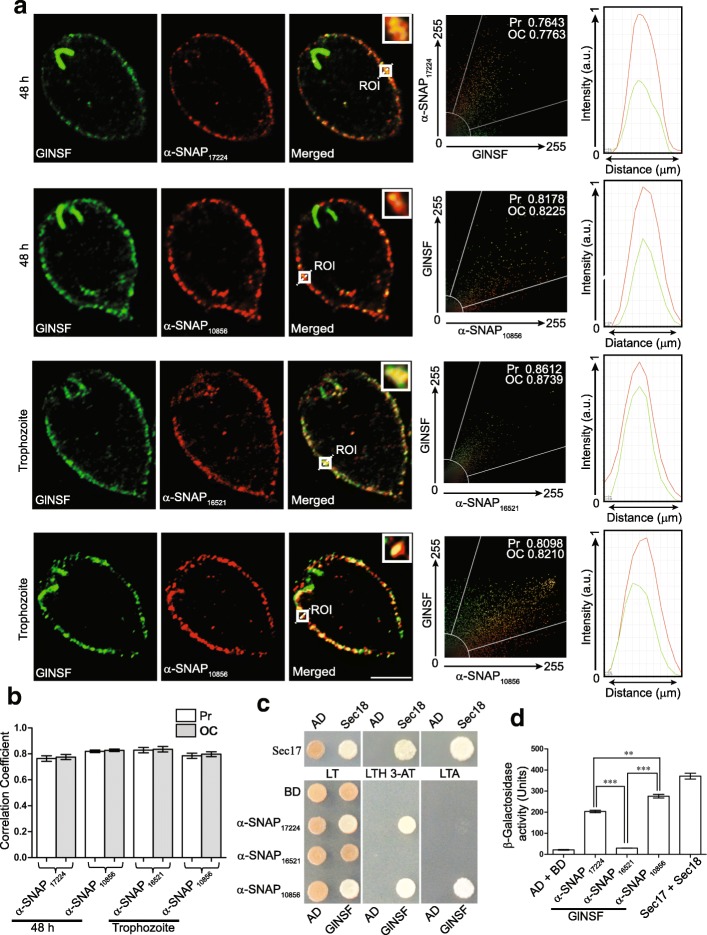
Colocalization and binary interaction between GlNSF and the giardial α-SNAPs. **a** Colocalization of GlNSF with α-SNAP_17224_ or α-SNAP_10856_ in 48 h encysting trophozoites, and with α-SNAP_16521_ or α-SNAP_10856_ in trophozoites. Insets depict magnification of the ROI (marked with a white box). The scattergram in each row indicates the analysis of colocalization between the two fluorophores over the entire z-stack by considering the pixels within the entire area occupied by the particular cell. The values for Pearson correlation coefficient (Pr) and overlap coefficient (OC) are indicated inside the scattergrams. The intensity plots at the extreme right indicate changes in the intensity of the red and green fluorescence signals across the diagonal of the ROI depicted by a dotted white line. **b** The bar graph denotes the mean Pr and OC calculated from the z-stacks of six independent images. **c** PJ69-4A cells were transformed with various combinations of constructs expressing fusion proteins with either Gal4 DNA binding domain (BD) or its activation domain (AD). Expression of the BD or AD alone served as negative controls. Transformants were spotted on YCM plates lacking leucine and tryptophan (LT), or leucine, tryptophan and histidine with 2.5 mM 3-AT (LTH 3-AT), or leucine, tryptophan and adenine (LTA). **d** β-galactosidase activity of the indicate transformants was quantified. Statistical significance of the difference in interaction between any two interacting pairs is indicated (**P < 0.01, ***P < 0.001). *Scale-bar*: **a**, 5 μm

Even though all the three paralogues function as α-SNAP in yeast, they exhibit heterogeneity in terms of their localization in *Giardia*. This scenario may arise if one or more of these paralogues do not function as an α-SNAP. Since α-SNAP and NSF assemble into the functional 20S complex that brings about SNARE unwinding, we examined if the three paralogues colocalize with the giardial NSF (GlNSF), which is encoded by the ORF 50803_114776. Immunolocalization of GlNSF in trophozoites was performed with an antibody (raised in rat) that specifically recognized this protein in giardial protein extract (Additional file 1: Figure S3b). It was observed that GlNSF localized to the PVs and the “brush border” structures associated with the part of the anterior flagella that is proximal to the basal bodies (Fig. 5a) [41]. This distribution persisted in 48 h encysting cells, with the enhanced signal at the brush borders. While none of the α-SNAPs localized to the brush borders, all of them exhibit a peripheral distribution in either trophozoites or encysting trophozoites (Fig. 4a). Hence for colocalization of the α-SNAPs with GlNSF, conditions were chosen where each of the α-SNAPs exhibit a predominantly peripheral distribution, 48 h encysting trophozoites for α-SNAP_17224_, trophozoites for α-SNAP_16521_, and both trophozoites and encysting trophozoites (48 h) for α-SNAP_10856_ (Fig. 5a). Under each of the conditions tested, all the three α-SNAPs showed significant colocalization with the GlNSF at the cell periphery, but not at the brush borders of the anterior flagella (Fig. 5a, Additional file 1: Figure S7). Both the Pr and the OC values indicate significant colocalization in multiple cells (Fig. 5b). Since all three α-SNAPs colocalize with GlNSF and each of them functionally complements the *sec17-1* temperature-sensitive allele (Fig. 3), it is likely that they function as α-SNAP in *Giardia*. Interestingly, barring α-SNAP_10856_, the two other α-SNAPs and GlNSF localize to regions where there is no overlap of the SNAP and NSF signals. These include the perinuclear regions for α-SNAP_17224_, the PDR for α-SNAP_16521_, and the brush borders for GlNSF. Thus, it seems likely that in addition to functioning in the 20S complex, each of these proteins also discharges additional cellular function(s).

### Difference in the interactions between GlNSF and each of the three α-SNAPs

Given that the three α-SNAP paralogues colocalize with GlNSF, we wanted to test whether each of these three proteins is capable of directly interacting with the latter. We used yeast two-hybrid to assess this binary interaction (Fig. 5c). It is known that yeast Sec17 physically interacts with its own NSF (Sec18) [18]. Consequently, these two proteins exhibit strong interaction in our two-hybrid assay as cells co-expressing Sec17 from the bait vector (pGBT9 with *TRP1* selection marker) and Sec18 from the prey vector (pGAD424 with *LEU2* selection marker), exhibited robust growth, not only on plates lacking histidine (LTH 3-AT), but also on plates lacking adenine (LTA); in comparison to the former, the later selection medium imposes greater stringency as only cells harboring strongly interacting bait-prey pairs can grow in the absence of exogenously-added adenine [42]. Results of the spot assay indicated that the interaction between GlNSF and α-SNAP_10856_ was comparable to that between Sec17 and Sec18 as yeast transformants expressing this giardial protein pair were able to grow on both LTH 3-AT and LTA plates (Fig 5c). However, estimation of the activity of the *LacZ* reporter gene, which provides a quantitative estimation of binary interaction, indicates that the interaction between the giardial proteins was weaker compared to that between the yeast proteins (Fig. 5d). Transformants expressing GlNSF and α-SNAP_17224_ exhibited growth on LTH 3-AT, but not on LTA plates (Fig. 5c). Even the β-galactosidase activity indicates that this giardial α-SNAP’s interaction with GlNSF was weaker compared to that between GlNSF and α-SNAP_10856_ (Fig. 5d). However, there does not appear to be any interaction between GlNSF and α-SNAP_16521_ as there is no growth on both LTA and LTH 3-AT plates. Even the color of the spot growing on plates lacking leucine and tryptophan (LT) is comparable to those of the negative control (Fig. 5c), as is the β-galactosidase activity of this transformant (Fig. 5d). Based on these observations there appears to be a lack of binary interaction between α-SNAP_16521_ and GlNSF; however, we cannot rule out an interaction between these two proteins *in vivo* where additional cellular factors may stabilize this interacting pair. Taken together, the results of the yeast two-hybrid assay indicate that GlNSF interacts differentially with the three α-SNAPs, with the strongest interaction taking place with α-SNAP_10856_, followed by that with α-SNAP_17224_, and very little or no interaction with α-SNAP_16521_. This observed difference in the interaction of the three paralogous proteins with GlNSF further underscores the fact that the three giardial α-SNAPs are likely to have undergone functional divergence in this protist.

### Oxidative stress induced relocalization of α-SNAPs

**Fig. 6:**
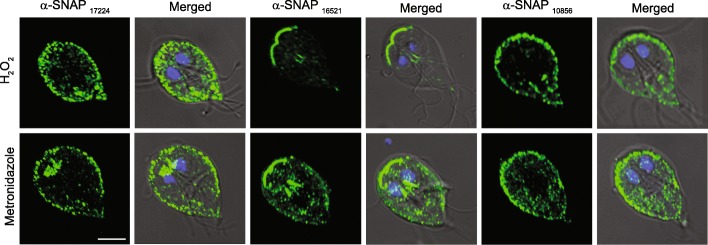
Localization of giardial α-SNAPs following oxidative stress. Localization of the three α-SNAPs in trophozoites exposed for 1 h to either 150 μm H_2_O_2_ (top row) or 1 μg/ml metronidazole (bottom row). *Scale-bar*: 5 μm

Based on the observed relocalization of α-SNAP_17224_ and α-SNAP_16521_ during encystation, we hypothesize that change in subcellular distribution of these two α-SNAPs may be part of *Giardia*’s response to changes in the external environment. If this hypothesis is true, then similar changes may occur when cells encounter oxidative stress. We chose oxidative stress in particular because existing literature indicates that *Giardia* is likely to have a unique mechanism for handling this stress. Not only is *Giardia* unable to tolerate elevated oxygen levels, it lacks several key components that are used by most eukaryotes to detoxify cellular reactive oxygen species [43]. Thus, while *Giardia* lacks enzymes such as catalase, glutathione peroxidase, and superoxide dismutase, its defense against oxidative stress includes noncanonical enzymes like an NADH oxidase, an NADH peroxidase, and a flavodiiron protein, to name a few [44]. To investigate if the presence of multiple paralogues of α-SNAP may be a part of this organism’s atypical mechanism to combat oxidative stress, we monitored the cellular distribution of these three proteins after inducing oxidative stress in trophozoites with two different agents, H_2_O_2_ (150 μM) and metronidazole (1 μg/ml) [45, 46]. Treatment with both reagents for a period of 1 h was sufficient to induce intracellular oxidative stress, as monitored by the conversion of DCFDA to the fluorescent DCF (Additional file 1: Figure S8). Following induction of oxidative stress, the relocalization of α-SNAP_17224_ and α-SNAP_16521_ was similar to that previously observed during the late stages of encystation (Compare Fig. 4a and Fig. 6; Additional file 3: Figure S10a and b, Additional file 2: Table S7). Thus, while α-SNAP_16521_ relocalized from the cell periphery to the PDRs, the signal for α-SNAP_17224_ moved from the perinuclear region to the cell periphery. However, unlike the change observed during encystation, a punctate cytoplasmic signal was also observed in both cases, with a cluster of puncta at the perinuclear region especially in case of α-SNAP_17224_ (Fig. 6). Consistent with the observed lack of change in subcellular distribution during encystation (Fig. 4a), α-SNAP_10856_ remained at the cell periphery even during oxidative stress (Fig. 6). However, like the other two α-SNAPs, cytoplasmic puncta were also observed in this case. Thus, in contrast to α-SNAP_10856_, the change in the distribution of α-SNAP_17224_ and α-SNAP_16521_ during both encystation and oxidative stress indicates that these two paralogues may have acquired additional functions during the course of evolution.

## Discussion

Although most eukaryotes encode only one α-SNAP, here we report that there are three paralogues in *Giardia*. These three genes are expressed in multiple morphological states of the parasite, thereby indicating that these three proteins are unlikely to discharge redundant functions in these different stages. Although α-SNAP_10856_ clusters with γ-SNAP sequences in the reconstructed phylogenetic tree, the results of the complementation analysis clearly indicate that it is functionally orthologous to α-SNAP (Fig. 3). The presence of three functional α-SNAP paralogues in a unicellular organism appears to be in stark contrast to most multicellular eukaryotes having only one homolog. Interestingly, the presence of multiple SNAPs has been documented in 47 protist genomes, including those of *Trichomonas*, *Entamoeba*, *Naegleria*, *Trypanosoma* and *Leishmania* and *in silico* analyses indicate that some of these putative proteins may be γ-SNAPs [47] (Dirk Fasshauer, personal communication). We have identified two SNAP paralogues in *S. salmonicida* and three in *Trepomonas* (Fig. 2). Thus, the presence of multiple SNAPs seems to be the norm for protists. Given that Protista represents a paraphyletic group, it is not possible to ascertain if a single gene duplication event gave rise to the expansion of the SNAP family, or if there were multiple independent such events. However, the fact that there is a difference in the number of SNAPs in closely-related diplomonad species, two in *S. salmonicida* and three in both *Giardia* and *Trepomonas*, indicates that later duplication events have also contributed to the expansion of this family of proteins in Protista.

While the results of the complementation assay indicate that each of the three paralogues are functionally analogous to Sec17 of yeast, the distinct subcellular localization of each protein in *Giardia* indicates that they do not perform redundant functions. In addition, results of the two-hybrid assay indicate that GlNSF had varying affinity for the three α-SNAPs. Given that GlNSF binds to α-SNAP_10856_ with the highest affinity, it may be hypothesized that the bulk of giardial *cis*-SNARE unpairing may be carried out by this paralogue. Its localization to the PV, where most of the SNAREs are present, lends support to this hypothesis [48]. Notably, this distribution does not change even during encystation, and after exposure to oxidative stress. Conversely, GlNSF has little or no interaction with α-SNAP_16521_ and this paralogue relocalizes to PDR region where neither GlNSF nor the SNAREs are documented to be present, suggesting that NSF attachment may not be necessary for the function(s) this protein discharges at the PDR. However, since α-SNAP_16521_ localizes to the PVs in trophozoites, its participation in SNARE complex disassembly cannot be ruled out.

One reason for the presence of such complexity in this unicellular protist may be the necessity to maintain *Giardia*’s asymmetric cell morphology. Unlike amoeboid protists, *Giardia* has a very unique tear-drop shape. The maintenance of this distinct asymmetric shape is likely to entail polarized vesicular trafficking to selective regions of the cell surface. However, the morphologically-simple endomembrane system of *Giardia* appears to lack key sorting stations, such as the Golgi. In the absence of readily-identifiable sorting compartments, *Giardia* may have evolved another system of determinants that allow selective targeting of vesicles to specific regions of the plasma membrane so that the shape of the cell is maintained. α-SNAP is already known to participate in such selective targeting in metazoans; it has been documented to enable polarized sorting to the apical surface of neuroepithelium as a single mutation causes missorting of apical proteins such as E-cadherin, β-catenin and F-actin [49]. The factors that play a role in giardial cell shape maintenance are difficult to trace as mutant hunts cannot be undertaken in this polyploid organism. Thus, it would be interesting to determine if there are any morphological changes following selective ablation of any one of these α-SNAPs.

The presence of α-SNAP_17224_ and α-SNAP_16521_ at cellular locations where NSF is not detectable is intriguing (Figs. 4a, 5a). One possibility is that α-SNAP may be performing NSF-independent functions at these locations. Existing literature indicates that α-SNAP has various NSF-independent roles [50]. An example of this is α-SNAP’s involvement in regulating calcium uptake *via* calcium release activated calcium (CRAC) channels [22]. Hexameric assemblies of the Orai1 protein at the PM forms the CRAC channel pores and this assembly process is regulated by α-SNAP as its deficiency results in loss of calcium selectivity of the CRAC channels, leading to dysregulated entry of sodium into the cell [51]. In another instance, α-SNAP interacts directly with the Bcl-2 family member, BNIP1, which is known to regulate the morphology of the ER [52]. Incidentally, we have also observed enhanced α-SNAP_17224_ signal at perinuclear regions, which are known to be occupied by the ER (Fig. 4a). The distribution of α-SNAP_16521_ at the PDRs is also indicative of a noncanonical role for this paralogue. Other proteins of *Giardia* with similar localization at the PDR include four proteins belonging to the Nek family of kinases (ORFs 5375, 92498, 16279 and 101534) and the catalytic subunits of protein phosphatase 2A (PP2Ac) and protein kinase A (PKAc) [53–55]. Nek proteins are documented to regulate ciliary function and assembly [56]. They also coordinate ciliary function with the cell cycle as they regulate the function of the centrioles, which serve both as basal bodies and microtubule-organizing centers [57]. Consistently, a study encompassing a large number of unikont and bikont organisms indicated that there is a direct correlation between the number of Nek genes encoded by a given genome and the presence of diving ciliated cell(s) in that particular organism [58]. *Giardia* has 56 active Neks, although its genome encodes 198; no other genome encodes such a large number of Neks and it is hypothesized that the expansion of this family of kinases is necessary to solve the challenges of coordinating the division of eight flagella with the cell cycle [58, 59]. Both PKAc and PP2Ac localize to the PDR in trophozoites [53, 54]. Since the signal for both proteins disappear simultaneously from the PDR of the anterior flagella early in encystation, it is postulated that they function in the same pathway in *Giardia* [53, 54]. PKAc is a known regulator of flagellar motility through its phosphorylation of dynein [60]. Thus, it is possible that since several other proteins that regulate flagellar function also localize to the PDRs, α-SNAP_16521_ may play a similar role. The difference in the α-SNAP_16521_ signal intensity at various PDRs may be because of differences in motility of each flagellar pair [61]. It may be noted that we have not detected the α-SNAPs at all the subcellular location where SNAREs of *Giardia* are known to localize [48]. For example, while gQb3 localizes to mitosomes, we have not observed similar distribution for any of the α-SNAPs. However, we cannot rule out the presence of a minor, and therefore undetectable, pool of any of the three α-SNAPs at other locations within the cell.

## Conclusions

The present study has uncovered the presence of three paralogues of α-SNAPs in *G. lamblia*. Expression of these three genes during multiple morphological states of the protist indicates that the function of each of these three α-SNAPs is required by the cell. Thus, these must be performing non-redundant functions. Antibodies raised against each of these three proteins were used to determine their subcellular distribution during different stages of the parasite’s life-cycle, and also upon exposure of the trophozoites to oxidative stress. These immunofluorescence experiments indicated a non-overlapping pattern of subcellular redistribution, without any accompanying change in the expression of the corresponding genes. In addition, two-hybrid assay established that these three paralogues have varying affinity for GlNSF. Taken together, it may be concluded that the three α-SNAP paralogues have undergone functional divergence in this protist. It was observed that α-SNAP_16521_ localizes to the PDRs associated with the anterior, posterolateral and caudal flagellar pairs. Given that nither GlNSF nor any of the SNAREs localize to the PDRs, it is possible that this paralogue performs an NSF-independent function. Thus, following duplication of the α-SNAP genes of *Giardia*, there may have been neofunctionalization of some of the paralogues.

## Methods

### Sequence analyses and secondary structure predictions

The protein sequences of *Giardia* SNAPs were curated from GiardiaDB and secondary structure predictions were carried out using iterative threading assembly refinement (I-TASSER) and Protein Homology/analogY Recognition Engine (Phyre2) servers [62, 63]. By default, both approaches used the crystal structure of *S. cerevisiae* Sec17 (PDB ID: 1QQE) as a template [27]. Based on these predictions, the secondary structural elements were marked on the multiple-sequence alignment that was generated with ClustalW, with editing in Jalview [64, 65]. Three-dimensional automatic threading models were generated in I-TASSER server, with the crystal structure of Sec17 as a template. The conserved positively charged residues were marked in Pymol [66].

### Phylogenetic analysis

Sequences of both α- and γ-SNAPs from organisms were curated using either NCBI (https://www.ncbi.nlm.nih.gov), UniProt (https://www.uniprot.org) or Eukaryotic Pathogen Database Resources (https://eupathdb.org) [67–69]. Domain analysis was performed with Pfam to ensure that the identified sequences contained a SNAP domain [70]. The curated sequences were used to reconstruct a maximum likelihood tree using MEGA7, with 100 bootstrap replicates [71].

### *Giardia lamblia* culture and *in vitro* encystation

Trophozoites of Assemblage A isolate Portland-1 were grown in slanted 15 ml culture tubes containing Diamond TYI-S-33 medium (pH 6.8) and the encystation was carried out as previously described [72, 73]. In order to obtain a pure preparation of cysts, 72 h post-induction of encystation, cells were harvested and incubated in distilled water at 4 °C, for 24 h for selective lysis of trophozoites that did not undergo encystation.

### Functional complementation

The temperature-sensitive strain RSY269 (MATα *ura3-52 his4-619 sec17-1*) was used for functional complementation [32]. *SEC17* and all the three *Giardia* α-SNAPs were PCR amplified with primers are listed in Additional file 2: Table S4. The PCR products were cloned under the control of a constitutive yeast promoter, in a 2 μm vector having *URA3* as a selectable marker (Additional file 2: Table S5). Each construct was individually transformed into RSY269. Resulting transformants were grown overnight in liquid YCM lacking uracil; serial dilutions of these cultures were spotted onto YCM plates lacking uracil and incubated at 30 and 37 °C.

### Polyclonal antibodies against giardial α-SNAPs and NSF

Each of the three giardial α-SNAPs was expressed and purified from BL21 (DE3) as previously described, except 0.2 mM IPTG was used [17]. The N-terminal region of GlNSF was also induced with the same concentration of IPTG but was purified from the pellet fraction, as previously described [35]. The primers used for cloning in pET32a are listed in Additional file 2: Table S4. The purified proteins were used to raise antibodies against α-SNAP_17224_ and α-SNAP_16521_ in rabbit, against α-SNAP_10856_ in mouse and against GlNSF in rat. All animal experiments adhered to the guidelines approved by the Institutional Animal Ethical Committee of Bose Institute (IAEC/BI/37/2015).

### Immunofluorescence and quantitative colocalization analyses

Immunofluorescence was performed in trophozoites, encysting trophozoites (time of encystation indicated in respective figures) and cysts as previously described [40]. Briefly, cells were harvested by chilling the culture tubes on ice, followed by centrifugation at 1000× *g* for 10 min. After washing with 1× PBS, the cells were fixed with 4% formaldehyde for 20 min at room temperature (RT). Following fixation, cells were harvested by centrifugation and treated with 0.1 M glycine for 5 min at RT. Subsequently, trophozoites and encysting trophozoites were permeabilized with 0.1% Triton X-100 in 1× PBS (v/v) for 15 min, followed by blocking with 2% BSA for 2 h at RT. Cysts were permeabilized with 0.2% Triton X-100 and 0.1% SDS for 15 min, before blocking with 2% BSA. For labeling of all the four giardial proteins, the respective primary antisera were used at 1:50 dilution in 0.2% BSA and incubated for overnight at 4 °C, with shaking. The following day, cells were washed thrice with 1× PBS and incubated with 1:400 dilution of any combination of the following secondary antibodies, as per requirement: Alexa Fluor 488-conjugated goat anti-rabbit, Alexa Fluor 488-conjugated goat anti-mouse, Alexa Fluor 594-conjugated goat anti-mouse, Alexa Fluor 594-conjugated goat anti-rabbit and Alexa Fluor 488-conjugated goat anti-rat. All secondary antibodies were procured from Abcam (Cambridge, UK). Before washing away secondary antibodies, cells incubated with DAPI at 1 μg/ml concentration for 15 min. Finally, cells washed three times with 1× PBS and resuspended in antifade medium (0.1% *p*-phenylenediamine in 90% glycerol). Samples were imaged with the 63× objective of a confocal laser scanning microscope (Leica TCS SP8, Wetzlar, Germany). 3D deconvolution and colocalization analyses were performed with Leica Application Suit X and images were assembled with Adobe Photoshop CS3 and Adobe Illustrator CS3.

### Statistical analysis

Statistical analyses for all the colocalization studies were performed with Leica Application Suit X (LAS X) software. The correlation coefficients, Pearson (Pr) and overlap (OC), values for each colocalization experiment were calculated on the basis of pixel-wise correlation between the signals emitted by the two fluorophores in each layer of six independent Z-stack. The Pr and OC values were plotted with GraphPad Prism 5.

### Real-time PCR

cDNA preparation and real-time PCR was performed as previously described, with primers designed against unique regions of giardial α-SNAPs (Additional file 2: Table S4**)** [35, 40]. PCR condition was as follows: initial denaturation at 95 °C for 5 min, second denaturation at 95 °C for 30 s, and annealing for 20 s at 56, 64.5 or 65.8 °C for SNAP_17224_, SNAP_16521_ and SNAP_10856_, respectively. The *C*_T_ values obtained for the three α-SNAP genes were normalized against the expression of ribosomal protein S5 (GL50803_12981), while the expression of the CWP1 gene served as positive control for encystation [37, 46]. Each experiment was performed in triplicate, with three technical replicates for each and data validation was done using two-tailed, paired t-test analysis in GraphPad Prism 5.

### Yeast two-hybrid assay

Yeast two-hybrid assay was performed using full length NSF and α-SNAPs of both yeast and *Giardia*, which were cloned in pGAD424 (prey vector having *LEU2* marker) and pGBT9 (bait vector having *TRP1* marker), respectively (Clonetech Laboratories, Mountain View, USA); the resulting fusion proteins had either the Gal4 activation domain (AD) or its DNA binding domain (BD), respectively (Additional file 2: Table S5) [74]. As per requirement, different pairs of the AD and BD constructs were co-transformed into the yeast strain PJ69-4A and the growth of each transformant, on YCM plates either lacking leucine, tryptophan and adenine (LTA) or lacking leucine, tryptophan and histidine, but having 2.5 mM 3-AT (LTH 3-AT), was monitored following incubation at 30 °C for 2 to 3 days [75]. The binary interaction between the various NSF and α-SNAP orthologues were also measured quantitatively by assessing the β-galactosidase activity by determining the nmol of o-nitrophenol formed from the hydrolysis of ONPG per min per mg of protein [76]. For this assay, experiments were performed in triplicate with two technical replicates for each sample. Results were statistically validated using a two-tailed, paired t-test in GraphPad Prism 5 software.

### Oxidative stress in *Giardia*

Trophozoites were grown to confluency. The old medium was replaced with freshly-prepared medium and cells were allowed to grow for another 2 h, prior to treatment with oxidative stress-inducing agents, either 150 μM H_2_O_2_ or 1 μg/ml metronidazole, for a period of 1 h, at 37 °C [45, 46]. To confirm intracellular ROS generation, cells were first harvested by chilling the tubes on ice, harvested by centrifugation at 1000× *g* for 10 min, washed thrice with warm PBS and treated with 2’,7’-dichlorodihydrofluoresceine diacetate (H_2_DCFDA) (Sigma D6883, St. Louis, USA) at a concentration of 1.5 μM, for 15 mins at 37 °C [45]. Finally, cells were fixed with 2% paraformaldehyde, washed thrice with PBS and observed under a confocal microscope.

## Additional files

Additional file 1: **Figure S1.** Circular dichroism spectrum of giardial SNAPs. Far-UV circular dichroism spectra of the three giardial SNAPs, in 20 mM sodium phosphate buffer at 16°C. Predicted helicity percentage, calculated by using the BeStSel server (bestsel.elte.hu/), is indicated in each spectrum. Percent helicity of Sec17, calculated on the basis of the crystal structure of Sec17 (1QQE), is 73%. **Figure S2.** Expression of the putative SNAPs during the life cycle of *Giardia*. The expression of the three putative SNAP orthologues of *Giardia* were determined by reverse transcriptase PCR, using the cDNA prepared from trophozoites, encysting cells and cysts. The primers used for this analysis are given in Additional file 2: Table S4. PCR products were visualized on 1.2% agarose gel. The length of all the PCR products for each SNAP correspond to the expected size. **Figure S3.** Specificities of the antibodies for giardial α-SNAPs and NSF. **a** Western blot with trophozoite extract and each of the three purified giardial α-SNAPs. Expression and purification of the proteins have been described in the Experimental Procedures section. The 6xHis-tag was removed from all three proteins prior to western blotting. The upper blot was incubated with anti-α-SNAP_17224_ antibody while the middle and lower blots were incubated with anti-α-SNAP_16521_ and anti-α-SNAP_10856_ antibodies, respectively. All antibodies were diluted 1000× prior to use. The presence of a ~34 kDa band in both the giardial extract and the corresponding overexpressed protein fraction in each blot indicates the specificity of that particular antibody. **b** Western blot with anti-GlNSF antibody using extracts of *Giardia* trophozoites and *E. coli* overexpressing GlNSF. Instead of the full-length protein, a stretch of 200-amino acids from the N-terminal segment of GlNSF was tagged with the 6xHis tag and expressed in *E. coli*. The expected size of this overexpressed protein is 39 kDa, while the size of the full-length GlNSF is 91 kDa. **Figure S4.** Localization of α-SNAP_10856_ to peripheral vesicles. **a** Colocalization of α-SNAP_10856_ with Lucifer yellow, a fluorescent dye that specifically stains acidic compartments, and, **b** a giardial PX domain-containing protein encoded by ORF GL50803_16548 and known to localize to the peripheral vesicles indicates that α-SNAP_10856_ localizes to the peripheral vesicles of trophozoites. The scattergram in each row indicates the analysis of colocalization between the two fluorophores, over the entire z-stack, by considering all the pixels within the whole area occupied by that cell. The values for Pearson correlation coefficient (Pr) and overlap coefficient (OC) are indicated within the respective scattergrams. The intensity plot at the right in the lower panel indicates changes in fluorescence intensity of the red and green signals across the dotted white line drawn across the ROI. *Scale-bars*: **a**, **b**, 5 μm. **Figure S5.** Immunolocalization of α-SNAP_17224_ and α-SNAP_16521_ to various subcellular structures. **a** Localization of α-SNAP_17224_ at the perinuclear region persisted after 30 h of encystation. The caret marks the perinuclear region. **b** α-SNAP_16521_ is located both at the peripheral vesicles (PV) and the paraflagellar dense rods (PDRs) of different flagella at 1.5 h post induction of encystation. However, at 4 h post induction, α-SNAP_16521_ is exclusively present at the PDRs, with no signal at the PVs. The triangle and arrow heads indicate PVs and the PDRs of different flagella, respectively. **c** Colocalization of α-tubulin (red) with α-SNAP_16521_ (green) in 16 h encysting trophozoites. The red and green signals are juxtaposed to each other at the anterior flagella, without any significant overlap. Cells were visualized with Alexa Fluor 488 and Cy5-conjugated secondary antibodies that bind to rabbit anti-α-SNAP_16521_ and mouse anti α-tubulin, respectively. **d** Two individual z-sections showing localization to the ventral disc periphery (left panel) and the PDRs (right panel). *Scale-bars*: **a**, **b**, **c**, **d**, 5 μm. **Figure S6.** Colocalization of α-SNAP_10856_ and a PX-domain containing protein in cysts. Both α-SNAP_10856_ and a PX-domain containing protein (GL50803_16548), a known lipid-interactor that localizes to vesicles, colocalize to cytoplasmic puncta in cysts. Carets mark some of the regions where there is considerable overlap of the red and green signals. The lower panels show regions of colocalization across the entire z-stack, with the left panel showing colocalization of the green signal (α-SNAP_10856_) on red (GL50803_16548) and the right panel shows the opposite, i.e. red on green. The intensity plot at the extreme right indicates changes in fluorescence intensity of the red and green signals across the dotted white line in the ROI. The values for Pearson (Pr) and overlap (OC) coefficient are given in the scatter plot. *Scale-bar*: 5 μm. **Figure S7.** Localization of GlNSF and α-SNAP_16521_. Localization of GlNSF (green) at the brush border (BB) and α-SNAP_16521_ (red) at the paraflagellar dense rods (PDRs) in 48 h encysting trophozoites. Each of the two fluorescent images has been individually merged with the DIC image to show the distribution of the fluorescent signals w.r.t. the flagella. *Scale-bar*: 5 μm. **Figure S8.** Detection of intracellular reactive oxygen species (ROS) by H_2_DCFDA. *Giardia* trophozoites were treated with either 150 μM H_2_O_2_ or 1μg/ml metronidazole for 1 h and intracellular ROS generation was monitored using H_2_DCFDA. The top row shows untreated cells with no fluorescence signal of DCF. Green fluorescence signal was observed in cells exposed to oxidative stress, with either H_2_O_2_ (middle row) or metronidazole (bottom row) due to the formation of reactive oxygen species. *Scale-bar*: 5 μm. (PPTX 3310 kb)

Additional file 2: **Table S1.** α-SNAP orthologues throughout the eukaryotes. **Table S2.** γ-SNAP orthologues throughout the eukaryotes. **Table S3.** SNARE binding residues of bovine α-SNAP and residues at analogous positions on Sec17 and giardial SNAPs. **Table S4.** Primer sequences. **Table S5.** List of constructs. **Table S6.** Percentage of cells exhibiting relocalization of the various α-SNAPs in different stages of encystation. **Table S7.** Percentage of cells exhibiting relocalization of the various α-SNAPs under oxidative stress. (DOCX 43 kb)

Additional file 3: **Figure S9.** Total number of *Giardia* cells with desired localization of three different α-SNAPs (α-SNAP17224 and α-SNAP10856 at the PVs, and α-SNAP16521 at the PDRs of different flagella) under, (**a**) H_2_O_2_ (150 μM) or (**b**) metronidazole (1 μg/ml) stress were counted from five different fields. The “banned” sign indicates cells that were excluded from the final count, either due to absence of signal or because the cells were deformed. Cells where a given α-SNAP localized to the region other than the desired one, were marked with “cross” sign. *Scale-bar*: 25 μm. **Figure S10.** Total number of *Giardia* cells with desired localization of three different α-SNAPs (α-SNAP17224 and α-SNAP10856 at the PVs, and α-SNAP16521 at the PDRs of different flagella) under, (**a**) H_2_O_2_ (150 μM) or (**b**) metronidazole (1 μg/ml) stress were counted from five different fields. The “banned” sign indicates cells that were excluded from the final count, either due to absence of signal or because the cells were deformed. Cells where a given α-SNAP localized to the region other than the desired one, were marked with “cross” sign. *Scale-bar*: 25 μm. (PDF 14472 kb)

## Abbreviations

3-AT: 3-amino-1,2,4-triazole
DAPI: 4’, 6-diamidino-2-phenylindole
DIC: Differential interference contrast
GlNSF: *Giardia lamblia* N-ethylmaleimide sensitive factor
NSF: N-ethylmaleimide sensitive factor
ONPG: Ortho-nitrophenyl-β-D-galactopyranoside
ORF: Open reading frame
PBS: Phosphate-buffered saline
PDR: Paraflagellar dense rod
PM: Plasma membrane
ROS: Reactive oxygen species
RT: Room temperature
SNAP: Soluble NSF attachment protein
SNARE: Soluble NSF attachment protein receptor
YCM: Yeast complete medium
