# Supplementary figures and images for: Multiple paralogues of α-SNAP in Giardia lamblia exhibit independent subcellular localization and redistribution during encystation and stress
Source: Parasit Vectors. 2018 Oct 4;11:539. doi: 10.1186/s13071-018-3112-1 (PMC6172762; doi:10.1186/s13071-018-3112-1)

## Slide 1
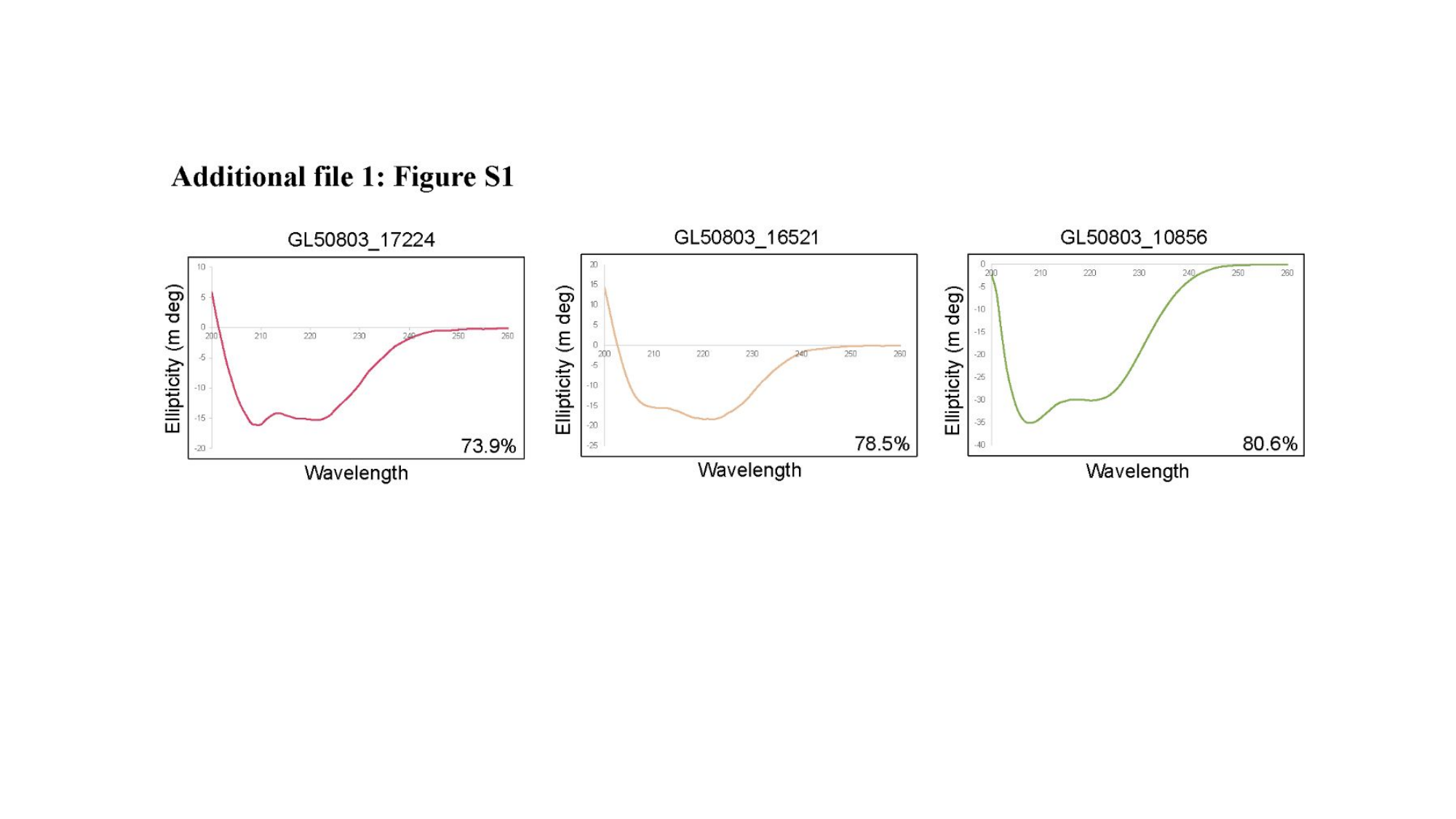

## Slide 2
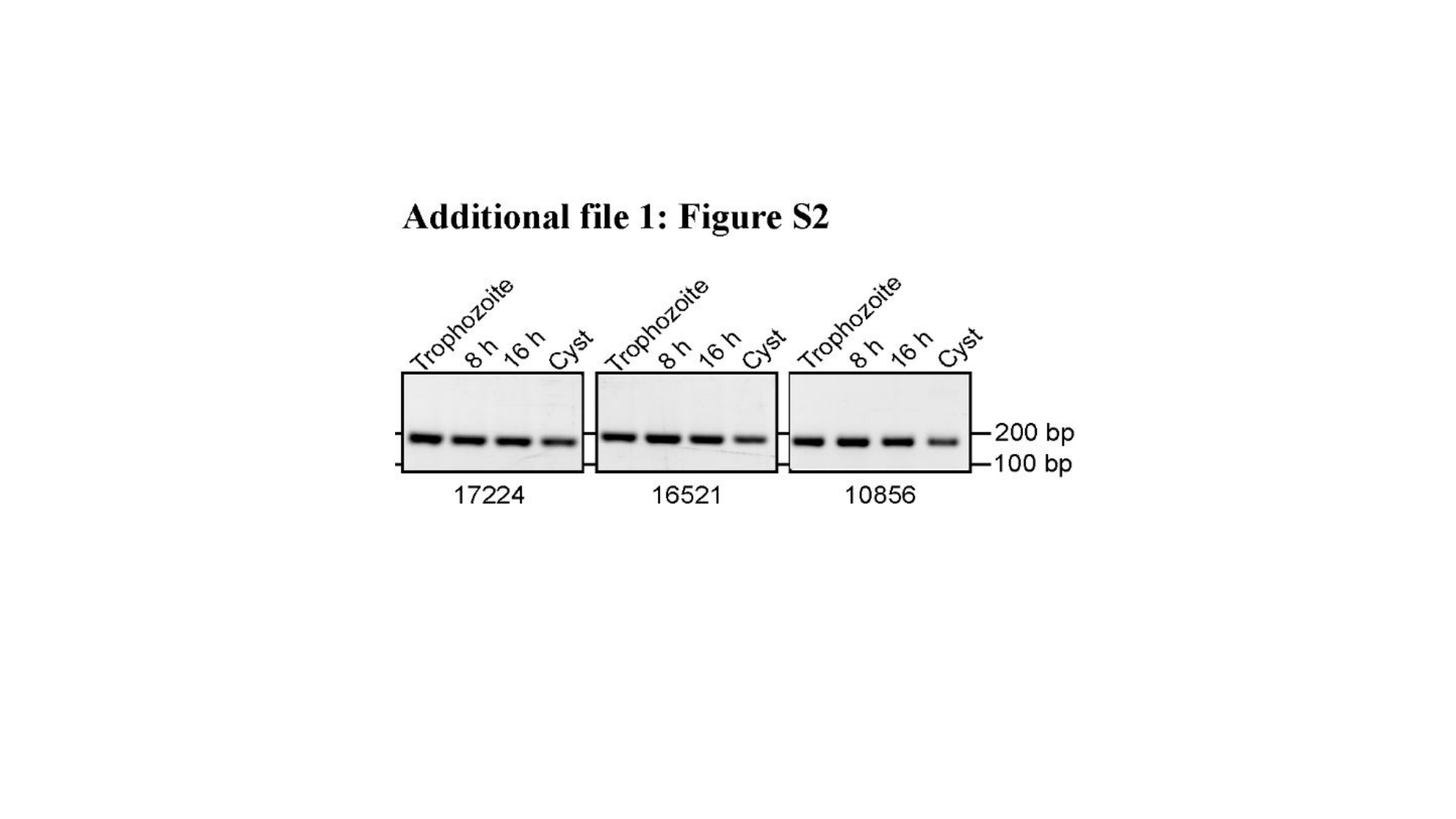

## Slide 3
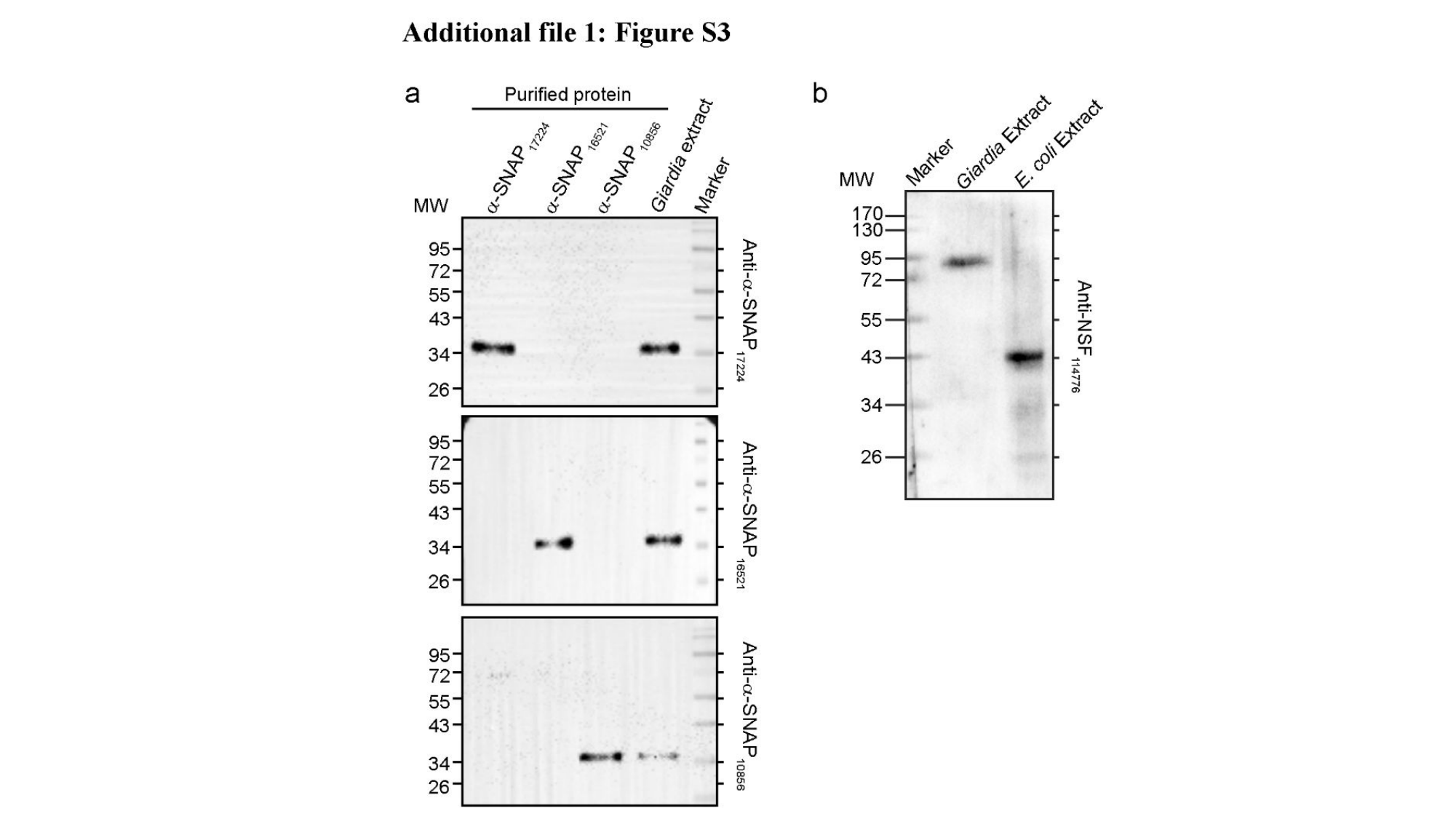

## Slide 4
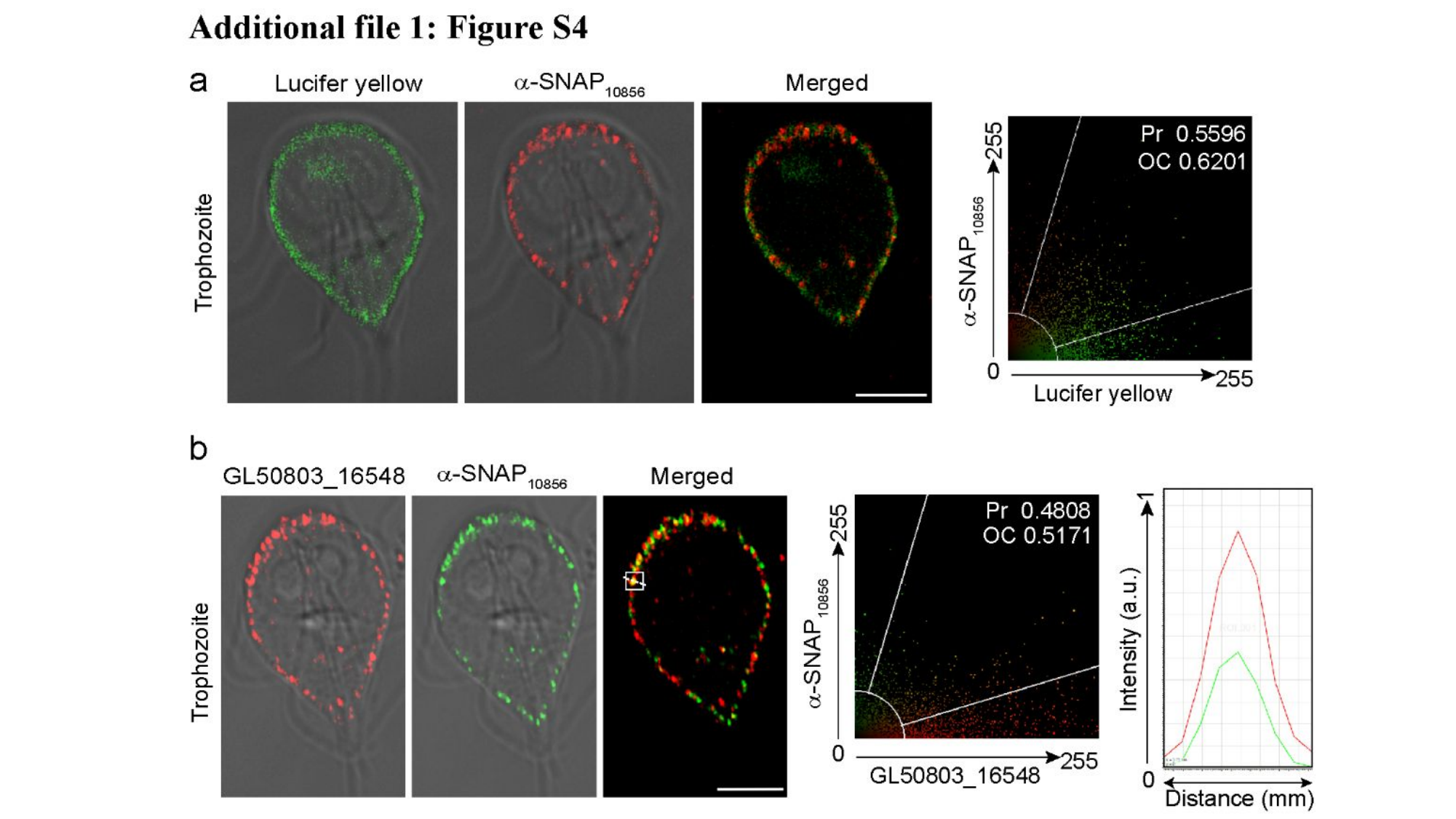

## Slide 5
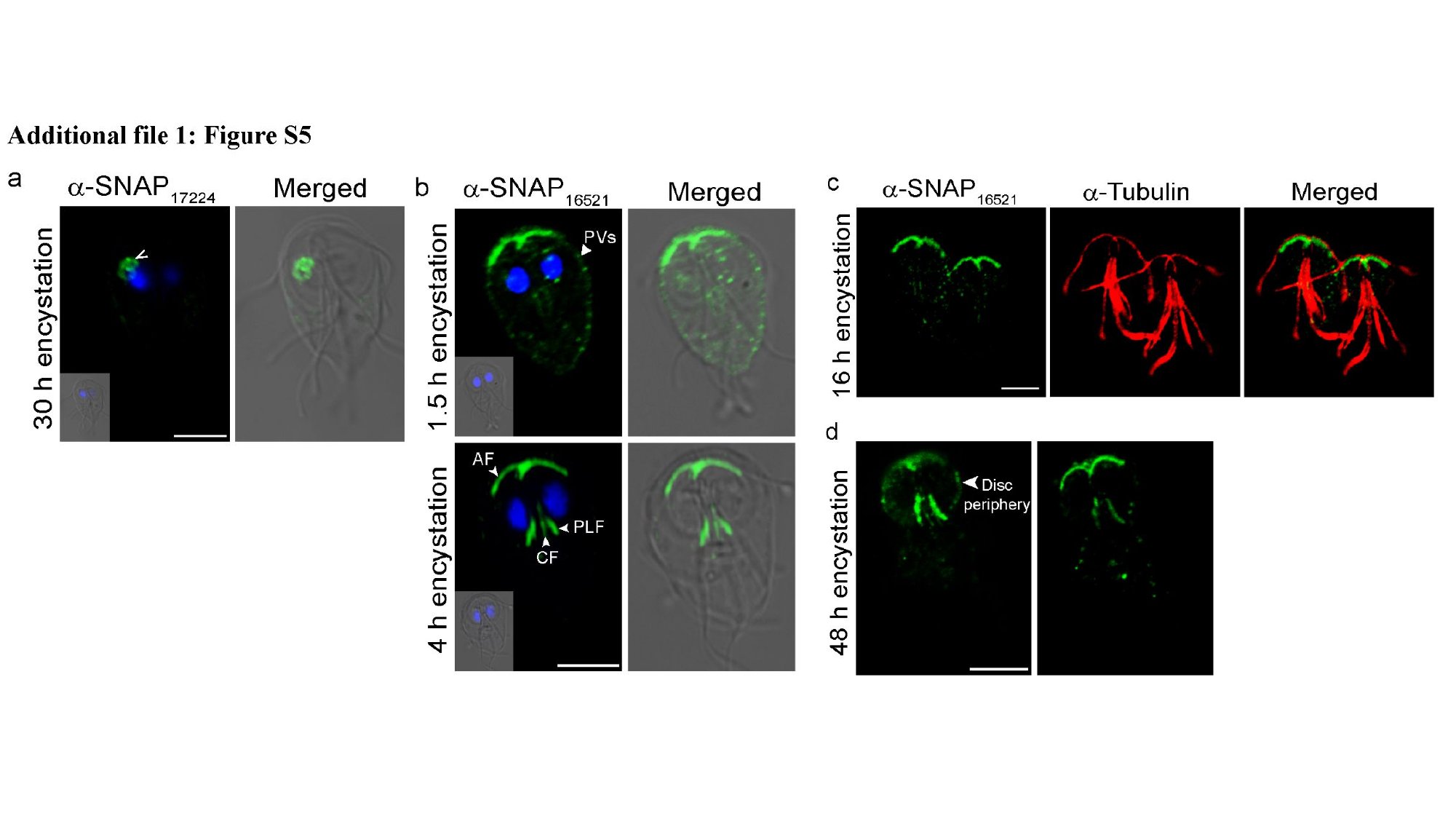

## Slide 6
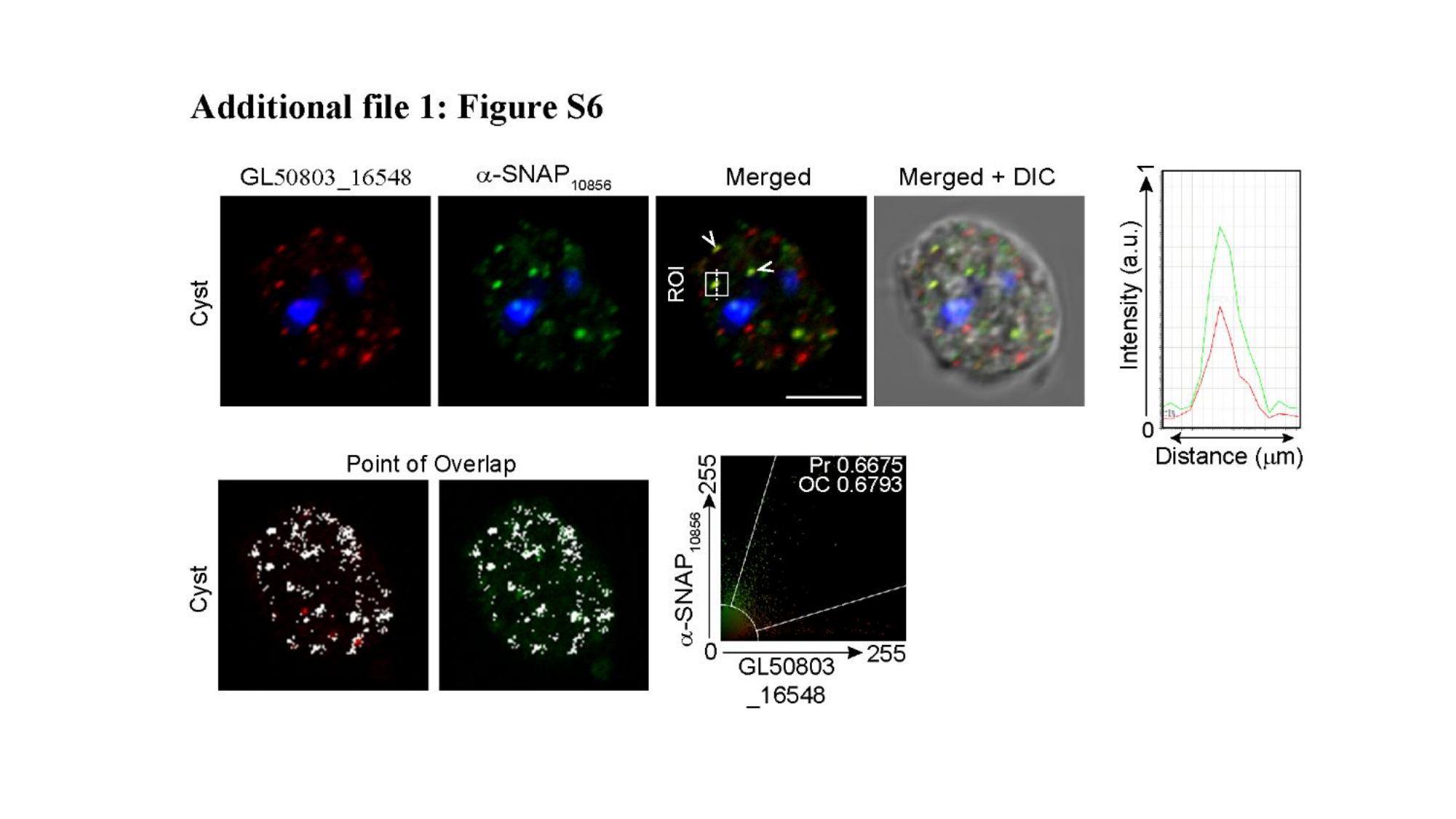

## Slide 7
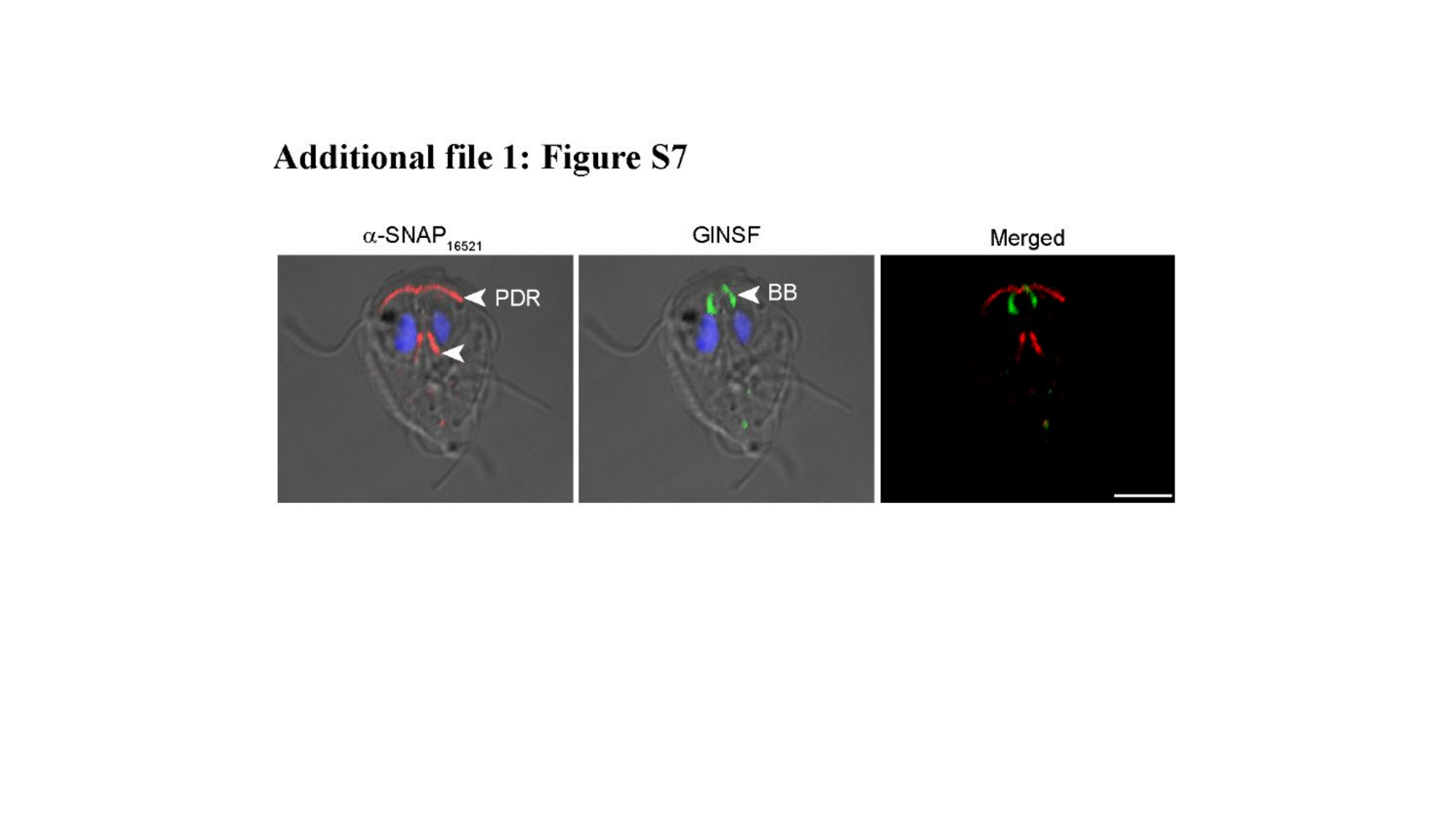

## Slide 8
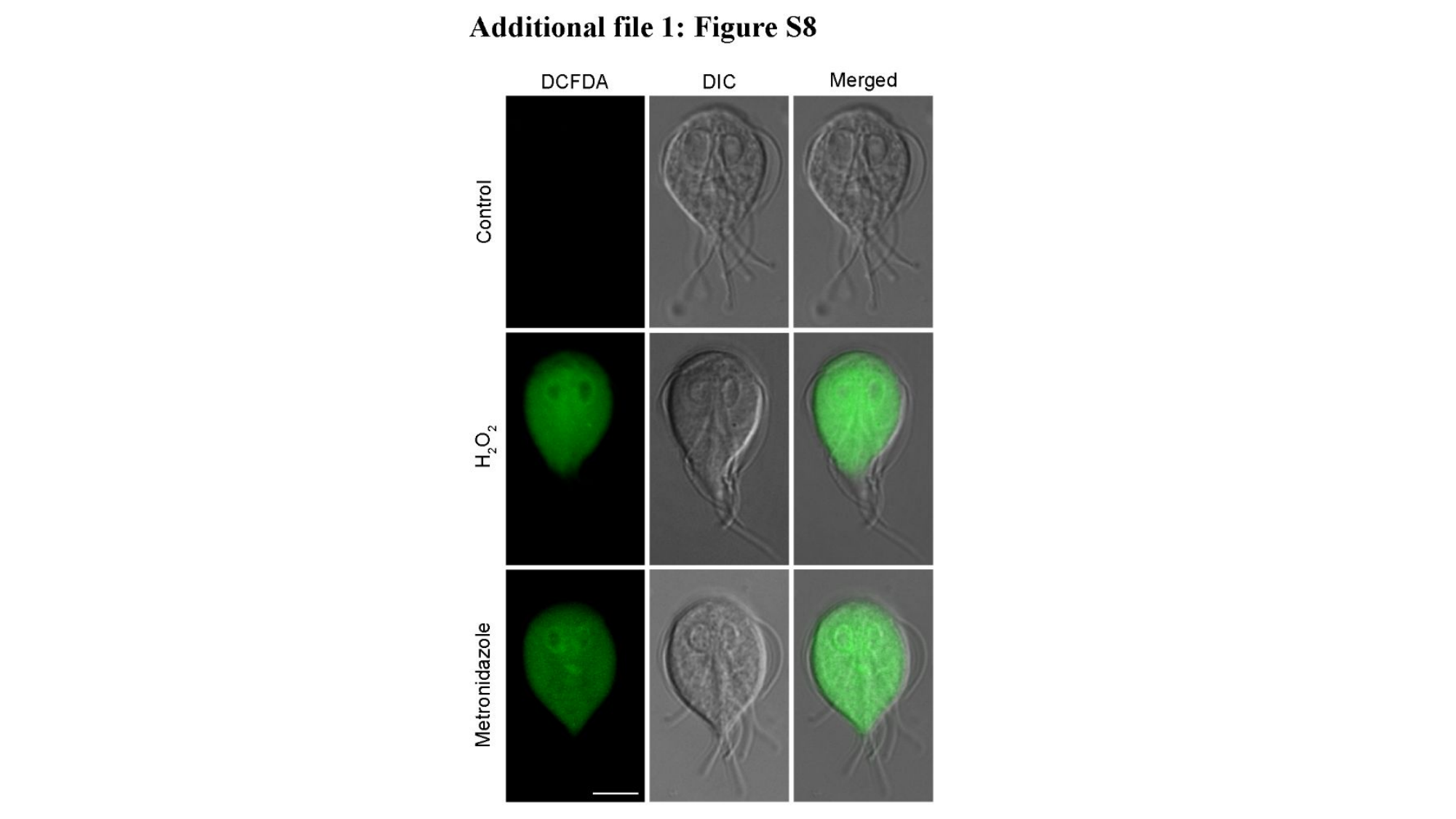

Supplement: Supplementary file 1 — Figure S1. Circular dichroism spectrum of giardial SNAPs. Far-UV circular dichroism spectra of the three giardial SNAPs, in 20 mM sodium phosphate buffer at 16°C. Predicted helicity percentage, calculated by using the BeStSel server (bestsel.elte.hu/), is indicated in each spectrum. Percent helicity of Sec17, calculated on the basis of the crystal structure of Sec17 (1QQE), is 73%. Figure S2. Expression of the putative SNAPs during the life cycle of Giardia. The expression of the three putative SNAP orthologues of Giardia were determined by reverse transcriptase PCR, using the cDNA prepared from trophozoites, encysting cells and cysts. The primers used for this analysis are given in Additional file 2: Table S4. PCR products were visualized on 1.2% agarose gel. The length of all the PCR products for each SNAP correspond to the expected size. Figure S3. Specificities of the antibodies for giardial α-SNAPs and NSF. a Western blot with trophozoite extract and each of the three purified giardial α-SNAPs. Expression and purification of the proteins have been described in the Experimental Procedures section. The 6xHis-tag was removed from all three proteins prior to western blotting. The upper blot was incubated with anti-α-SNAP17224 antibody while the middle and lower blots were incubated with anti-α-SNAP16521 and anti-α-SNAP10856 antibodies, respectively. All antibodies were diluted 1000× prior to use. The presence of a ~34 kDa band in both the giardial extract and the corresponding overexpressed protein fraction in each blot indicates the specificity of that particular antibody. b Western blot with anti-GlNSF antibody using extracts of Giardia trophozoites and E. coli overexpressing GlNSF. Instead of the full-length protein, a stretch of 200-amino acids from the N-terminal segment of GlNSF was tagged with the 6xHis tag and expressed in E. coli. The expected size of this overexpressed protein is 39 kDa, while the size of the full-length GlNSF is 91 kDa. Figure S4. Locali [file 13071_2018_3112_MOESM1_ESM.pptx]

$\alpha$ -SNAPs in encystation

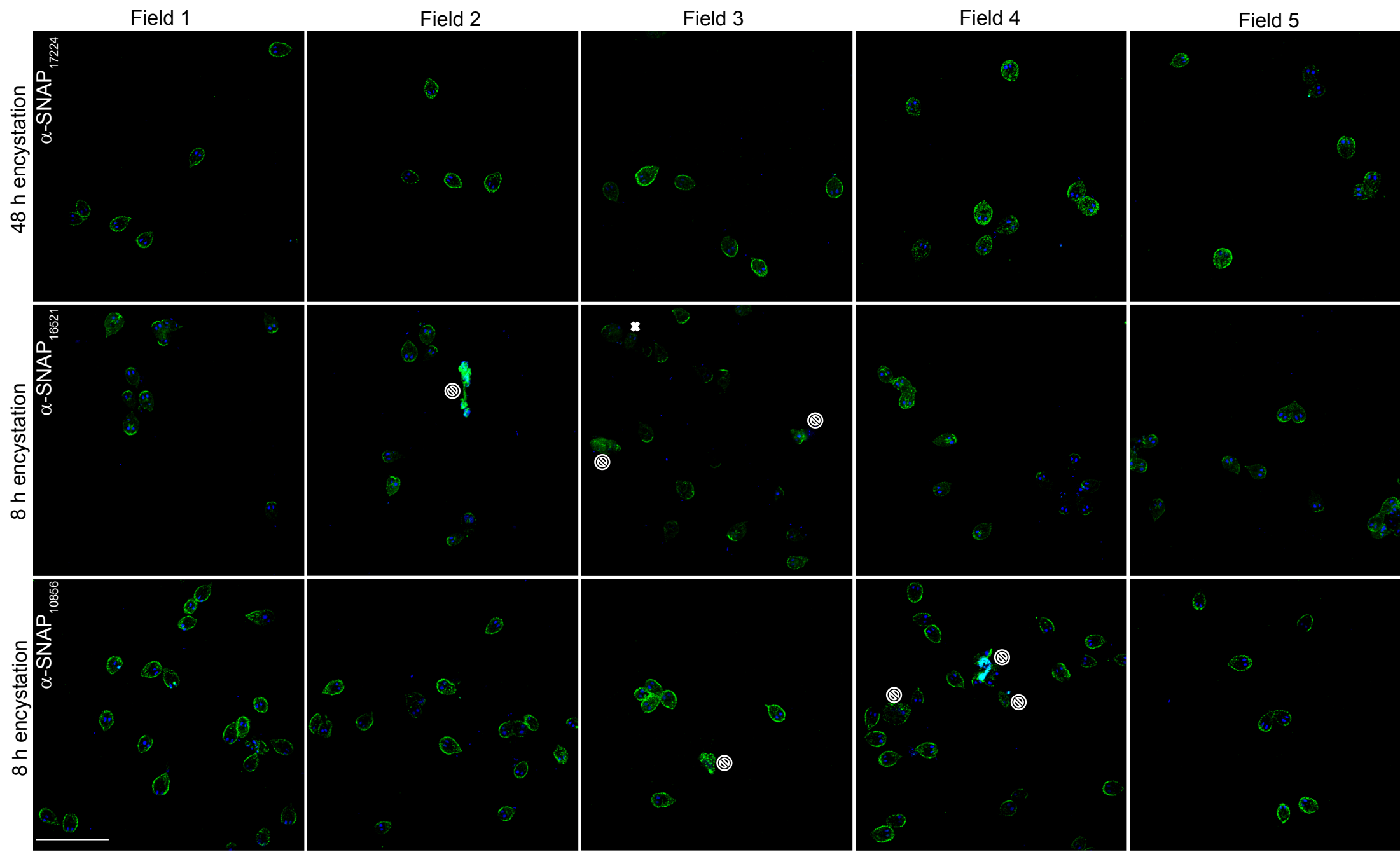

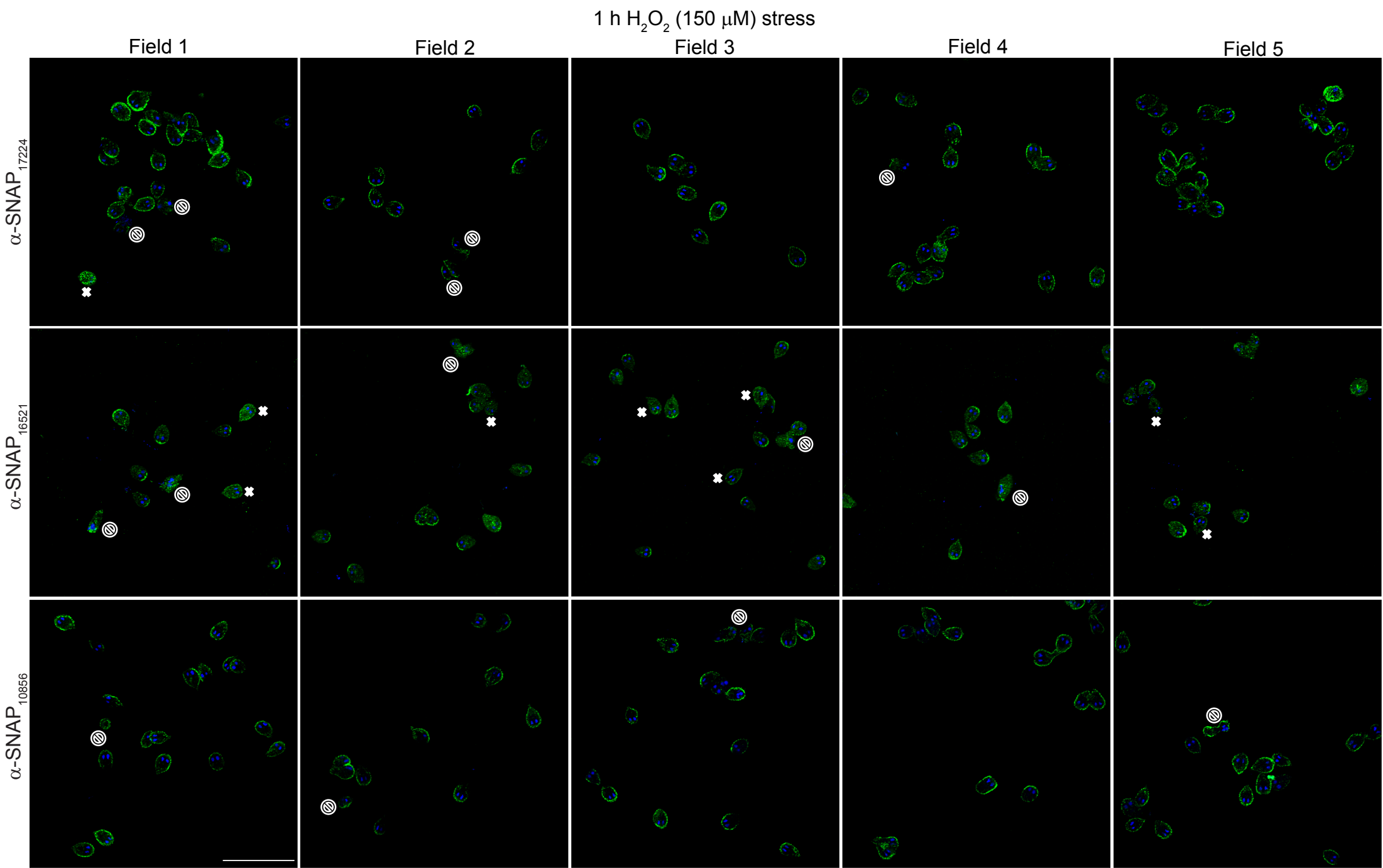

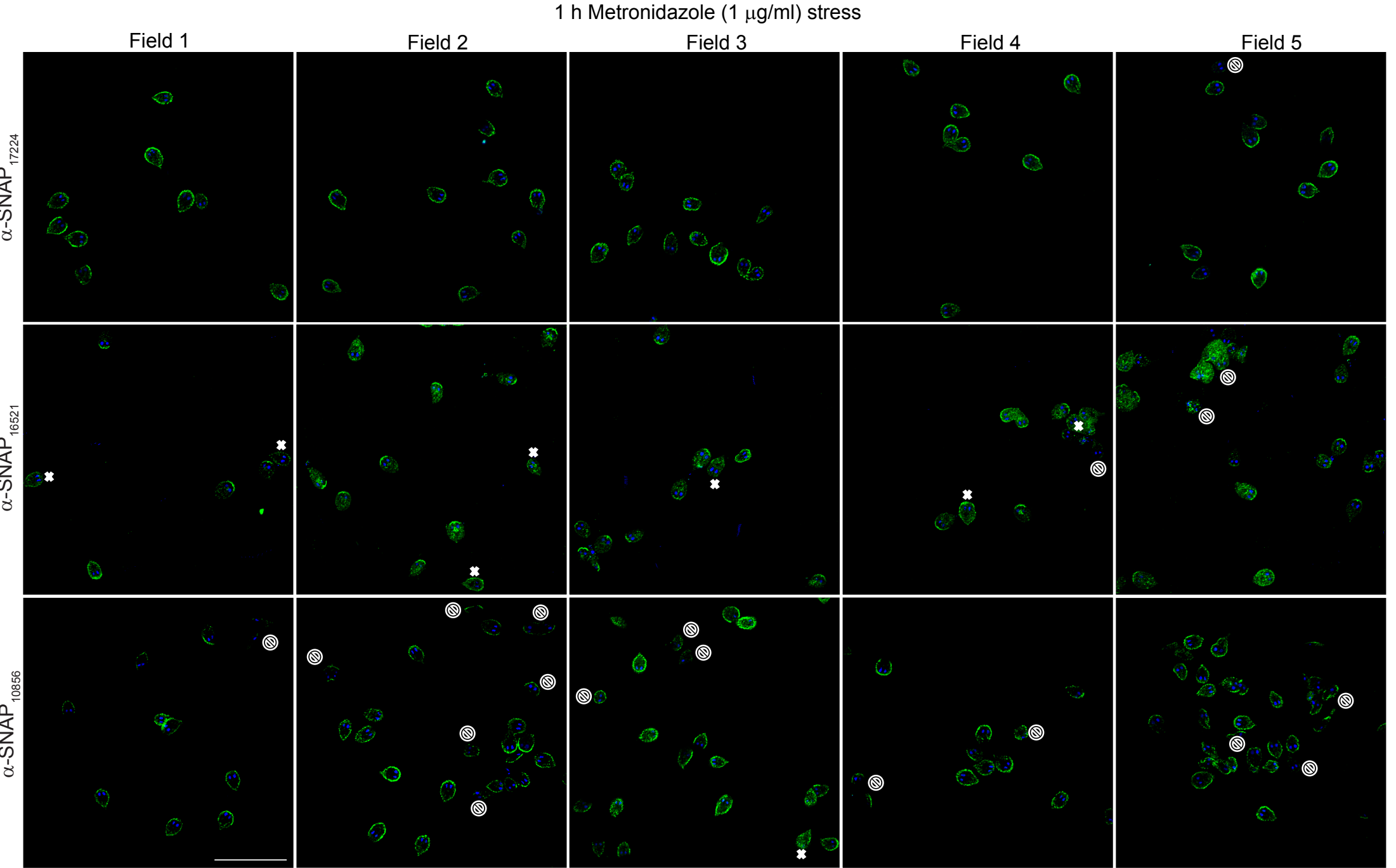

Supplement: Supplementary file 3 — Figure S9. Total number of Giardia cells with desired localization of three different α-SNAPs (α-SNAP17224 and α-SNAP10856 at the PVs, and α-SNAP16521 at the PDRs of different flagella) under, (a) H2O2 (150 μM) or (b) metronidazole (1 μg/ml) stress were counted from five different fields. The “banned” sign indicates cells that were excluded from the final count, either due to absence of signal or because the cells were deformed. Cells where a given α-SNAP localized to the region other than the desired one, were marked with “cross” sign. Scale-bar: 25 μm. Figure S10. Total number of Giardia cells with desired localization of three different α-SNAPs (α-SNAP17224 and α-SNAP10856 at the PVs, and α-SNAP16521 at the PDRs of different flagella) under, (a) H2O2 (150 μM) or (b) metronidazole (1 μg/ml) stress were counted from five different fields. The “banned” sign indicates cells that were excluded from the final count, either due to absence of signal or because the cells were deformed. Cells where a given α-SNAP localized to the region other than the desired one, were marked with “cross” sign. Scale-bar: 25 μm. (PDF 14472 kb) [file 13071_2018_3112_MOESM3_ESM.pdf]
