# Supplementary material for: Multiple paralogues of α-SNAP in Giardia lamblia exhibit independent subcellular localization and redistribution during encystation and stress
Source: Parasit Vectors. 2018 Oct 4;11:539. doi: 10.1186/s13071-018-3112-1 (PMC6172762; doi:10.1186/s13071-018-3112-1)
Supplement: Supplementary file 2 — Table S1. α-SNAP orthologues throughout the eukaryotes. Table S2. γ-SNAP orthologues throughout the eukaryotes. Table S3. SNARE binding residues of bovine α-SNAP and residues at analogous positions on Sec17 and giardial SNAPs. Table S4. Primer sequences. Table S5. List of constructs. Table S6. Percentage of cells exhibiting relocalization of the various α-SNAPs in different stages of encystation. Table S7. Percentage of cells exhibiting relocalization of the various α-SNAPs under oxidative stress. (DOCX 43 kb) [file 13071_2018_3112_MOESM2_ESM.docx]

| ***Arabidopsis***  ***thaliana* 1 & 2** | ***Homo sapiens*** | ***Bos taurus*** | ***Rattus norvegicus*** | ***Drosophila melanogaster*** | ***Saccharomyces***  ***cerevisiae*** | ***Spironucleus***  ***salmonicida***  **1 & 2** | ***Giardia lamblia***  **GL50803_17224**  **GL50803_16521**  **GL50803_10856** |
| --- | --- | --- | --- | --- | --- | --- | --- |
| ***Arabidopsis***  ***thaliana* 1**  ***Arabidopsis***  ***thaliana* 2** | **40.1/65.2**  **20.4/34.2** | **40.5/65.9**  **20.6/34.7** | **40.5/65.6**  **20.8/34.7** | **43.1/66.7**  **20.8/33.5** | **34.1/53.2**  **18.3/30.1** | **26.7/48.2**  **22.2/39.2**  **15.9/28**  **12.5/24.3** | **28.3/49.7**  **24.3/43.7**  **23.9/37.9**  **18.7/30.8**  **14.5/26.9**  **11.5/18.8** |
|  | ***Homo sapiens*** | **98.6/99.7** | **98.0/99.3** | **60.9/79.1** | **34.1/55.2** | **25.6/47.9**  **22.2/40.5** | **26.9/46**  **23.9/47.1**  **20.8/37.8** |
|  |  | ***Bos taurus*** | **98.0/99.7** | **62/79.5** | **33.8/54.8** | **25.9/47.9**  **22.2/40.8** | **27.2/46.3**  **23.9/47.1**  **21.8/38.6** |
|  |  |  | ***Rattus norvegicus*** | **61.1/79.1** | **34.1/55.2** | **25.6/47.9**  **22.2/40.8** | **26.9/45.6**  **23.9/47.1**  **21.8/38.6** |
|  |  |  |  | ***Drosophila melanogaster*** | **33/55.9** | **27.1/48.4**  **22/39.3** | **27.3/42.9**  **24.4/45.7**  **21/34.3** |
|  |  |  |  |  | ***Saccharomyces***  ***cerevisiae*** | **21.1/37**  **21.2/36** | **25.6/40.9**  **19.7/32.8**  **21.1/34.6** |
|  |  |  |  |  |  | ***Spironucleus***  ***salmonicida* 1**  ***Spironucleus***  ***salmonicida* 2** | **39.6/55.6**  **28.4/51.3**  **18.9/34.6**  **23.3/38.5**  **14.2/27.4**  **23.4/43.8** |

**Additional file 2: Table S1.** α-SNAP orthologues throughout the eukaryotes

**Additional file 2:** **Table S2.** γ-SNAP orthologues throughout the eukaryotes

| ***Arabidopsis thaliana*** | ***Homo sapiens*** | ***Bos taurus*** | ***Rattus norvegicus*** | ***Drosophila melanogaster*** | ***Spironucleus salmonicida* 1& 2** | ***Giardia lamblia***  **GL50803_17224**  **GL50803_16521**  **GL50803_10856** |
| --- | --- | --- | --- | --- | --- | --- |
| ***Arabidopsis thaliana*** | **23.2/43.9** | **22.2/41.8** | **16.0/32.6** | **23.2/45.1** | **16.1/26.3**  **19.3/35.5** | **16.9/29.2**  **13.9/25.1**  **20.5/37.9** |
|  | ***Homo sapiens*** | **90.5/93.3** | **71.5/72.4** | **37.8/60.3** | **19.3/32.6**  **21.5/34.1** | **23.1/38.6**  **14.4/24.4**  **20.1/37.3** |
|  |  | ***Bos Taurus*** | **66.8/68.3** | **37.2/57.7** | **20.3/34.8**  **22.4/34.3** | **22.8/34.2**  **14.1/23.7**  **17.5/32.5** |
|  |  |  | ***Rattus norvegicus*** | **24.4/40.9** | **16.2/27.5**  **14.8/23.4** | **14.4/24**  **15.4/26.2**  **13.7/27.8** |
|  |  |  |  | ***Drosophila melanogaster*** | **20.8/37.8**  **19.8/32.4** | **24.0/39.2**  **17.3/27.4**  **18.0/32.7** |
|  |  |  |  |  | ***Spironucleus salmonicida* 1**  ***Spironucleus salmonicida* 2** | **39.6/55.6**  **28.4/43.8**  **18.9/34.6**  **23.3/38.5**  **14.2/27.4**  **23.4/43.8** |

**Additional file 2:** **Table S3.**  SNARE binding residues of bovine α-SNAP and residues at analogous positions on Sec17 and giardial SNAPs

| ***Bos taurus*** | **Sec17** | **GL50803_17224** | **GL50803_16521** | **GL50803_10856** |
| --- | --- | --- | --- | --- |
| R47 | R50 | K38 | K37 | - |
| K53 | R48 | K44 | K43 | K54 |
| K56 | - | K47 | K46 | - |
| K94 | K88 | K84 | K83 | K94 |
| R116 | R110 | R107 | - | K123 |
| K122 | R115 | K113 | K112 | R129 |
| K163 | K159 | R153 | R152 | K159 |
| K167 | K163 | H158 | R157 | R160 |
| Y200 | W196 | F192 | K194 | Y202 |
| K203 | K199 | R195 | F198 | K205 |

**Additional file 2:** **Table S4**. Primer sequences

| Sl No. | Primer Sequence (5’ to 3’) | Purpose |
| --- | --- | --- |
| 1. | ATGACTCAGGCAAGTGAA | Forward primer for RT of Glα-SNAP_17224_ |
| 2. | GGACCTTCTCAAAGAGGAA | Reverse primer for RT of Glα-SNAP_17224_ |
| 3. | GCTGGCAAAAGAATCTGAGG | Forward primer for RT of Glα-SNAP_16521_ |
| 4. | TGCGGCTTCTCATGGGAA | Reverse primer for RT of Glα-SNAP_16521_ |
| 5. | GCCTTTCAAATGTACACATCG | Forward primer for RT of Glα-SNAP_10856_ |
| 6. | CTCGGGTGAAAGCAATCTGT | Reverse primer for RT of Glα-SNAP_10856_ |
| 7. | CAAGGATCCGCTTCAGGCAGATGTCTGACTACG | Forward primer for cloning Glα-SNAP_17224_ for complementation |
| 8. | AATGCTGCAGGCTAAGTCGGCACTGATGTGACATTG | Reverse primer for cloning Glα-SNAP_17224_ for complementation |
| 9. | CAAGGATCCGAGGTTCCATCCAGACGAAACATCG | Forward primer for cloning Glα-SNAP_16521_ for complementation |
| 10. | AATGCTGCAGACTCAAGGTCGTTGACGTTGTCC | Reverse primer for cloning Glα-SNAP_16521_ for complementation |
| 11. | TATCCCGGGGTCACAATCATCCTCTGAAGGGCTC | Forward primer for cloning Glα-SNAP_10856_ for complementation |
| 12. | CGTGGATCCCACTGCTCAAAGGTACTTCTCGGCATC | Reverse primer for cloning Glα-SNAP_10856_ for complementation |
| 13. | ACTGGATCCGTAATGATAAGAAGTGGGCAGCACCG | Forward primer for cloning *SEC17* for complementation |
| 14. | ATCTAGTCGACGATGGCTTCAACTTCGCTCTTGC | Reverse primer for cloning *SEC17* for complementation |
| 15. | CGTGGAATTCGGCTTCAGGCAGATGTCTGACTACG | Forward primer for cloning Glα-SNAP_17224_ in pET32a |
| 16. | GCTAAAGCTTGCTAAGTCGGCACTGATGTGACATTG | Reverse primer for cloning Glα-SNAP_17224_ in pET32a |
| 17. | GTGAGGTACCATGAGTTATGCAAAGCAGGCGGAG | Forward primer for cloning Glα-SNAP_16521_ in pET32a |
| 18. | CATTGTCGACCGTTGACGTTGTCCTCGTTCATCC | Reverse primer for cloning Glα-SNAP_16521_ in pET32a |
| 19. | CAGAATTCGCACTTCATCCGTGTCAGATGC | Forward primer for cloning Glα-SNAP_10856_ in pET32a |
| 20. | GACAAGCTTTCACTGCTCAAAGGTACTTCTCGGC | Reverse primer for cloning Glα-SNAP_10856_ in pET32a |
| 21. | CAGAGAATTCATGTTCCATTCCACACCTTTGTCC | Forward primer for cloning GlNSF_114776_ in pET32a |
| 22. | GTAGTCGACGTTACCAGTTCAGCAGACTCTTGTG | Reverse primer for cloning GlNSF_114776_ in pET32a |
| 23. | CGTGGAATTCGGCTTCAGGCAGATGTCTGACTACG | Forward primer for cloning Glα-SNAP_17224_ in pGBT9 |
| 24. | GCTAGGATCCGCTAAGTCGGCACTGATGTGACATTG | Reverse primer for cloning Glα-SNAP_17224_ in pGBT9 |
| 25. | GAGGGGATCCACAACATGAGTTATGCAAAGCAGGCGG | Forward primer for cloning Glα-SNAP_16521_ in pGBT9 |
| 26. | CCTTGTCGACCGTTGACGTTGTCCTCGTTCATC | Reverse primer for cloning Glα-SNAP_16521_ in pGBT9 |
| 27. | CAGAATTCGCACTTCATCCGTGTCAGATGC | Forward primer for cloning Glα-SNAP_10856_ in pGBT9 |
| 28. | GACGGATCCTCACTGCTCAAAGGTACTTCTCG | Reverse primer for cloning Glα-SNAP_10856_ in pGBT9 |
| 29. | CGGAGAATTCATGTTCCATTCCACACCTTTGTCC | Forward primer for cloning GlNSF_114776_ in pGAD424 |
| 30. | AGTAGTCGACGTTACCAGTTCAGCAGACTCTTGTG | Reverse primer for cloning GlNSF_114776_ in pGAD424 |
| 31. | CGAGGATCCAAATGTCAGACCCTGTAGAGTT | Forward primer for cloning Sec17 in pGBT9 |
| 32. | CGAGTGTCGACCGTAAGTATATGCCGTTCATAAC | Reverse primer for cloning Sec17 in pGBT9 |
| 33. | GGCGGATCCACAGTATGTTCAAGATACCTGGTTTTG | Forward primer for cloning Sec18 in pGAD424 |
| 34. | GCTGTGTCGACGGTTCACTAGATCCTTTCTGATTGG | Reverse primer for cloning Sec18 in pGAD424 |

Note: The restriction sites used in the primers are underlined

**Additional file 2:** **Table S5.** List of constructs

| Plasmid Name | Cloned gene | Description | Primers used |
| --- | --- | --- | --- |
| pSPD1 | *Gl*α-SNAP_17224_ | *Gl*α-SNAP_17224_ (pRS426 *URA3*) | 7 and 8 |
| pSPD2 | *Gl*α-SNAP_16521_ | *Gl*α-SNAP_16521_ (pRS426 *URA3*) | 9 and 10 |
| pSPD3 | *Gl*α-SNAP_10856_ | *Gl*α-SNAP_10856_ (pRS426 *URA3*) | 11 and 12 |
| pSPD4 | *ScSEC17* | *ScSEC17* (pRS426 *URA3*) | 13 and 14 |
| pSPD5 | *Gl*α-SNAP_17224_ | *Gl*α-SNAP_17224_ (pET32a) | 15 and 16 |
| pSPD6 | *Gl*α-SNAP_16521_ | *Gl*α-SNAP_16521_ (pET32a) | 17 and 18 |
| pSPD7 | *Gl*α-SNAP_10856_ | *Gl*α-SNAP_10856_ (pET32a) | 19 and 20 |
| pSPD8 | *Gl*NSF_114776_ | *Gl*NSF_N-terminal (pET32a) | 21 and 22 |
| pSPD9 | *Gl*α-SNAP_17224_ | *Gl*α-SNAP_17224_ (pGBT9) | 23 and 24 |
| pSPD10 | *Gl*α-SNAP_16521_ | *Gl*α-SNAP_16521_ (pGBT9) | 25 and 26 |
| pSPD11 | *Gl*α-SNAP_10856_ | *Gl*α-SNAP_10856_ ( pGBT9) | 27 and 28 |
| pSPD12 | *Gl*NSF_114776_ | *Gl*NSF_114776_ ( pGAD424) | 29 and 30 |
| pSPD13 | *ScSEC17* | *ScSEC17* (pGBT9) | 31 and 32 |
| pSPD14 | *ScSEC18* | *ScSEC18* (pGAD424) | 33 and 34 |

**Additional file 2:** **Table S6.** Percentage of cells exhibiting relocalization of the various α-SNAPs in different stages of encystation

| **Protein** | **Stage of encystation** | **Field**  **Number** | **Number of cells in the field** | **Number of cells showing α-SNAP relocalization** |
| --- | --- | --- | --- | --- |
| α-SNAP_17224_ | 48 h | 1 | 06 | 06 |
|  |  | 2 | 04 | 04 |
|  |  | 3 | 06 | 06 |
|  |  | 4 | 08 | 08 |
|  |  | 5 | 07 | 07 |
|  |  | **Total: 31 31 (100%)** | | |
| α-SNAP_16521_ | 8 h | 1 | 10 | 10 |
|  |  | 2 | 07 | 07 |
|  |  | 3 | 15 | 14 |
|  |  | 4 | 11 | 11 |
|  |  | 5 | 10 | 10 |
|  |  | **Total: 53 52 (98.11%)** | | |
| α-SNAP_10856_ | 8 h | 1 | 20 | 20 |
|  |  | 2 | 17 | 17 |
|  |  | 3 | 06 | 06 |
|  |  | 4 | 19 | 19 |
|  |  | 5 | 07 | 07 |
|  |  | **Total: 69 69 (100%)** | | |

| **Protein** | **Oxidative stress** | **Field**  **Number** | **Number of cells in the field** | **Number of cells showing α-SNAP relocalization** |
| --- | --- | --- | --- | --- |
| α-SNAP_17224_ | 150 µM H_2_O_2_ for 1 h | 1 | 21 | 20 |
|  |  | 2 | 07 | 07 |
|  |  | 3 | 08 | 08 |
|  |  | 4 | 15 | 15 |
|  |  | 5 | 21 | 21 |
|  |  | **Total: 72 71 (98.61%)** | | |
| α-SNAP_16521_ | 150 µM H_2_O_2_ for 1 h | 1 | 08 | 06 |
|  |  | 2 | 11 | 10 |
|  |  | 3 | 12 | 09 |
|  |  | 4 | 06 | 06 |
|  |  | 5 | 10 | 08 |
|  |  | **Total: 47 39 (82.97%)** | | |
| α-SNAP_10856_ | 150 µM H_2_O_2_ for 1 h | 1 | 17 | 17 |
|  |  | 2 | 10 | 10 |
|  |  | 3 | 12 | 12 |
|  |  | 4 | 14 | 14 |
|  |  | 5 | 15 | 15 |
|  |  | **Total: 68 68 (100%)** | | |
|  | | | | |
| **Protein** | **Oxidative stress** | **Field**  **Number** | **Number of cells in the field** | **Number of cells showing α-SNAP relocalization** |
| α-SNAP_17224_ | 1 µg/ml Metronidazole for 1 h | 1 | 09 | 09 |
|  |  | 2 | 09 | 09 |
|  |  | 3 | 11 | 11 |
|  |  | 4 | 07 | 07 |
|  |  | 5 | 09 | 09 |
|  |  | **Total: 45 45 (100%)** | | |
| α-SNAP_16521_ | 1 µg/ml Metronidazole for 1 h | 1 | 06 | 04 |
|  |  | 2 | 11 | 09 |
|  |  | 3 | 11 | 10 |
|  |  | 4 | 09 | 07 |
|  |  | 5 | 15 | 15 |
|  |  | **Total: 52 45 (86.53%)** | | |
| α-SNAP_10856_ | 1 µg/ml Metronidazole for 1 h | 1 | 09 | 09 |
|  |  | 2 | 16 | 16 |
|  |  | 3 | 16 | 15 |
|  |  | 4 | 08 | 08 |
|  |  | 5 | 21 | 21 |
|  |  | **Total: 70 69 (98.57%)** | | |

**Additional file 2: Table S7.** Percentage of cells exhibiting relocalization of the various α-SNAPs under oxidative stress

| **Protein** | **Oxidative stress** | **Field**  **Number** | **Number of cells in the field** | **Number of cells showing α-SNAP relocalization** |
| --- | --- | --- | --- | --- |
| α-SNAP_17224_ | 150 µM H_2_O_2_ for 1 h | 1 | 21 | 20 |
|  |  | 2 | 07 | 07 |
|  |  | 3 | 08 | 08 |
|  |  | 4 | 15 | 15 |
|  |  | 5 | 21 | 21 |
|  |  | **Total: 72 71 (98.61%)** | | |
| α-SNAP_16521_ | 150 µM H_2_O_2_ for 1 h | 1 | 08 | 06 |
|  |  | 2 | 11 | 10 |
|  |  | 3 | 12 | 09 |
|  |  | 4 | 06 | 06 |
|  |  | 5 | 10 | 08 |
|  |  | **Total: 47 39 (82.97%)** | | |
| α-SNAP_10856_ | 150 µM H_2_O_2_ for 1 h | 1 | 17 | 17 |
|  |  | 2 | 10 | 10 |
|  |  | 3 | 12 | 12 |
|  |  | 4 | 14 | 14 |
|  |  | 5 | 15 | 15 |
|  |  | **Total: 68 68 (100%)** | | |
|  | | | | |
| **Protein** | **Oxidative stress** | **Field**  **Number** | **Number of cells in the field** | **Number of cells showing α-SNAP relocalization** |
| α-SNAP_17224_ | 1 µg/ml Metronidazole for 1 h | 1 | 09 | 09 |
|  |  | 2 | 09 | 09 |
|  |  | 3 | 11 | 11 |
|  |  | 4 | 07 | 07 |
|  |  | 5 | 09 | 09 |
|  |  | **Total: 45 45 (100%)** | | |
| α-SNAP_16521_ | 1 µg/ml Metronidazole for 1 h | 1 | 06 | 04 |
|  |  | 2 | 11 | 09 |
|  |  | 3 | 11 | 10 |
|  |  | 4 | 09 | 07 |
|  |  | 5 | 15 | 15 |
|  |  | **Total: 52 45 (86.53%)** | | |
| α-SNAP_10856_ | 1 µg/ml Metronidazole for 1 h | 1 | 09 | 09 |
|  |  | 2 | 16 | 16 |
|  |  | 3 | 16 | 15 |
|  |  | 4 | 08 | 08 |
|  |  | 5 | 21 | 21 |
|  |  | **Total: 70 69 (98.57%)** | | |
